# Validating the Health Benefits of Coffee Berry Pulp Extracts in Mice with High-Fat Diet-Induced Obesity and Diabetes

**DOI:** 10.3390/antiox13010010

**Published:** 2023-12-20

**Authors:** Khawaja Muhammad Imran Bashir, Joo Wan Kim, Hye-Rim Park, Jae-Kyoung Lee, Beom-Rak Choi, Jae-Suk Choi, Sae-Kwang Ku

**Affiliations:** 1 Department of Seafood Science and Technology, The Institute of Marine Industry, Gyeongsang National University, Tongyeong 53064, Republic of Korea; imran.bashir@lstme.org; 2 German Engineering Research and Development Center for Life Science Technologies in Medicine and Environment, Busan 46742, Republic of Korea; 3 Department of Companion Animal Health, Daegu Haany University, Gyeongsan 38610, Republic of Korea; 4 Nutracore Co., Ltd., Suwon 16514, Republic of Korea; 5 Department of Anatomy and Histology, College of Korean Medicine, Daegu Haany University, Gyeongsan 38610, Republic of Korea; 6 CNS Pharm Korea Co., Ltd., Seoul 04043, Republic of Korea; 7 Department of Food Regulatory Science, College of Science and Technology, Korea University Sejong Campus, Sejong 30019, Republic of Korea

**Keywords:** antioxidant, *Coffea arabica* L., coffee berry pulp, diabetic obese mice, high-fat-diet, type 2 diabetes

## Abstract

The effects of coffee (*Coffea arabica* L.) berry pulp extracts (CBP extracts) on the improvement of diabetes, obesity, and non-alcoholic fatty liver disease (NAFLD) were evaluated using various in vitro antioxidant activity assays and through a high-fat diet-induced mild diabetic obese mouse model. After an 84-day oral administration of CBP extracts (400–100 mg/kg), bioactivities were evaluated. The in vitro analysis showed the highest DPPH^●^ scavenging activity of 73.10 ± 4.27%, ABTS^●^ scavenging activity of 41.18 ± 1.14%, and SOD activity of 56.24 ± 2.81%, at a CBP extract concentration of 1000 µg/mL. The in vivo analysis of the CBP extracts showed favorable and dose-dependent anti-obesity, anti-diabetic, NAFLD, nephropathy, and hyperlipidemia refinement effects through hepatic glucose enzyme activity, 5′-AMP-activated protein kinase (AMPK) up-regulation, antioxidant activity, lipid metabolism-related gene expression, and pancreatic lipid digestion enzyme modulatory activities. This study shows that an appropriate oral dosage of CBP extracts could function as a potent herbal formulation for a refinement agent or medicinal food ingredient to control type 2 diabetes and related complications.

## 1. Introduction

Obesity is a significant risk factor for various metabolic diseases, including cardiovascular diseases, hypertension, low-grade inflammation, and type 2 diabetes [1,2,3]. The excessive consumption of fatty acids leads to triglyceride (TG) accumulation in various tissues, accompanied by an increase in lipolysis. Simultaneously, this triggers a rise in circulating fatty acids in the blood, inducing insulin resistance in adipocytes. Consequently, there is fat accumulation in the muscle, pancreas, and liver. Insulin resistance further induces the overproduction of fatty acid transport and binding proteins, negatively impacting insulin-mediated glucose metabolism in non-adipocytes, particularly muscle cells. Long-term exposure to free fatty acids in the pancreas impairs insulin secretion through a mechanism known as lipotoxicity [4]. This phenomenon, combined with high free fatty acid accumulation in the liver, induces insulin resistance, leading to the release of a substantial amount of glucose from the liver [5]. Hepatocyte TG accumulation contributes to non-alcoholic fatty liver disease (NAFLD), resulting in fat accumulation, secondary steatohepatitis, and fibrosis [6]. Maintaining a balance between fat degradation and synthesis in hepatocytes emerges as a crucial therapeutic target to curb the induction of NAFLD and insulin resistance associated with metabolic syndrome [7].

Due to the adverse effects associated with currently recommended therapeutic agents for metabolic syndromes [8], there is a concerted effort to develop natural product-based alternative medicines with enhanced effectiveness and minimal side effects [9,10,11]. Several natural products have demonstrated anti-obesity and anti-diabetic effects owing to their antioxidant and anti-inflammatory properties [9,10,11,12,13]. A daily intake of 4–6 cups of coffee has been suggested to provide enough active ingredients with physiological activity [14]. Notably, coffee berries (*Coffea arabica* L.) have been noted for their higher antioxidant levels [15,16], antibacterial properties [17], and effects in combating obesity and improving insulin resistance [18,19]. Key components of coffee, such as chlorogenic acids, caffeine, trigonelline, and diterpenes, including cafestol, have exhibited physiological activities beyond caffeine [20,21,22,23]. Caffeic acid metabolites, such as caffeoylquinic acids (CQAs), are known for their potent antioxidant and anti-obesity effects [18,19], and cafestol has recently demonstrated blood sugar regulatory effects [22,23]. However, to the best of our knowledge, there are limited studies confirming the anti-obesity and type 2 diabetes treatment effects of coffee cherry pulp. Considering the escalating cases of obesity and type 2 diabetes, there is a critical need to explore alternative compounds from natural sources for treating diabetes and related complications.

Utilizing a high-fat diet (HFD) in mice has proven effective in inducing diabetic obesity, comparable to human metabolic syndrome [10,11,24,25]. Consequently, the HFD-fed mouse model serves as a valuable tool for evaluating the efficacy of functional foods and drug candidates [9,10,11,12,13]. In this study, we investigated the pharmacological activity of Coffee Berry Pulp extracts (CBP extracts) from *C. arabica* L. in an HFD-fed mouse model. This study aimed to assess the impact of CBP extracts on obesity and related metabolic syndromes, including hyperglycemia, insulin resistance, NAFLD, and diabetic nephropathy. Our findings were compared with those of mice administered metformin, serving as a control [9,10,11,12,13,25].

## 2. Materials and Methods

### 2.1. Sample

The powdered coffee berry pulp extracts (CBP extracts) from *Coffea arabica* L. were generously provided by Nutracore Co., Ltd., Suwon, Republic of Korea. The light brown CBP extracts exhibited a solubility of up to 40 mg/mL in distilled water and was stored at −20 °C until use. A portion of the powdered CBP extracts was preserved as a sample in the herbarium of the Medical Research Center for Herbal Convergence on Liver Disease, Daegu Haany University, Gyeongsan, Republic of Korea (Reference No.: CBP2022BPK01).

### 2.2. High-Performance Liquid Chromatography Analyses (HPLC)

To conduct HPLC analyses, 250 mg of CBP extracts was dissolved in 5 mL of tertiary distilled water, and methanol was added to increase the volume to 25 mL. The dissolved CBP extracts were filtered using a 0.45 µm filter disc, and 10 µL of the sample was injected into the Agilent HPLC 1200-DAD system (Agilent Technologies, Inc., Santa Clara, CA, USA). The system was equipped with a Kromasil C18 column (4.6 mm × 100 mm, 10 μm; Nouryon, Bohus, Sweden) and a UV-vis absorbance detector. Mobile phases comprised 0.05% trifluoroacetic acid in water (A) and methanol (B) with a flow rate set at 1.0 mL/min. The proportions of both mobile phases were as follows: 0 min A (95%): B (5%), 30 min A (65%): B (35%), and 35 min A (0%): B (100%). Absorbance values were recorded at 330 nm, and chlorogenic acid (Sigma-Aldrich, St. Louis, MO, USA) served as the standard for quantitative analysis.

### 2.3. In Vitro Assessment of Antioxidant Activity

#### 2.3.1. DPPH^●^ Scavenging Activity

The 2,2-diphenyl-1-picrylhydrazyl radical (DPPH^●^; Sigma-Aldrich Co., St. Louis, MO, USA) scavenging activity of CBP extracts was determined according to the method of Kuda et al. [26] with slight modifications. CBP extracts at concentrations ranging from 0 to 1000 µg/mL were used, and L-ascorbic acid (0.25 μg/mL; Sigma-Aldrich) and HPLC-grade water (J. T. Baker, Chemical Co., Ltd., Corporate Parkway, PA, USA) served as positive and negative controls, respectively. Absorbance at 517 nm was measured using the Infinite 200 Pro microplate reader (TECAN Group Ltd., Männedorf, Switzerland), and DPPH^●^ scavenging activity was calculated using Equation (1).

DPPH^●^ scavenging activity (%) = [(A_control_ − A_blank1_) − (A_sample_ − A_blank2_)/(A_control_ − A_blank1_)] × 100 (1)

here, A represents the absorbance measured at 517 nm, blank_1_ signifies the HPLC-grade water blank, and blank_2_ designates the sample blank.

#### 2.3.2. ABTS^●^ Scavenging Activity

The 2,2′-azino-di-3-ethylbenzthiazoline sulfonic acid radical (ABTS^●^; Sigma-Aldrich) scavenging activity of CBP extracts followed the method of Re et al. [27] with slight adjustments. CBP extracts at concentrations from 0 to 1000 µg/mL were used, and L-ascorbic acid (10 μg/mL; Sigma-Aldrich) and HPLC-grade water (J. T. Baker) served as positive and negative controls, respectively. Absorbance at 734 nm was measured using the Infinite 200 Pro microplate reader (TECAN), and ABTS^●^ scavenging activity was calculated using Equation (2).

ABTS^●^ scavenging activity (%) = [1 − (A_sample_/A_control_)] × 100 (2)

here, A signifies the absorbance of samples and controls measured at 734 nm.

#### 2.3.3. SOD Activity

In vitro superoxide dismutase (SOD) activity was assessed using the SOD-assay kit-WST (Dojindo Laboratories, Kumamoto, Japan) following the manufacturer’s instructions. CBP extracts at concentrations ranging from 0 to 1000 µg/mL were used, and L-ascorbic acid (100 μg/mL; Sigma-Aldrich) and HPLC-grade water (J. T. Baker) served as positive and negative controls, respectively. Absorbance at 450 nm was measured using the Infinite 200 Pro microplate reader (TECAN), and the SOD activity was determined using Equation (3).

SOD activity (%) = [(A_blank1_ − A_blank3_) − (A_sample_ − A_blank2_)]/(A_blank1_ − A_blank3_) × 100 (3)

here, A_blank1_ signifies the absorbance of the inhibitor blank containing HPLC-grade water, WST kit reagent solution, and SOD enzyme working solution. A_blank2_ designates the sample blank containing the sample solution and WST kit reagent solution, while A_blank3_ represents the reagent blank consisting of HPLC-grade water, WST kit reagent solution, and the WST kit dilution buffer.

### 2.4. Animal Model

A total of 80 six-week-old SPF/VAF CrlOri:CD1 (ICR; ♀) mice were procured from Orient Bio, Seungnam, Republic of Korea. Only the mice (*n* = 60) showing a constant weight gain after ten days of acclimatization and one week of HFD feeding were selected for this study. The selected 60 animals were randomly assigned to a total of six groups (*n* = 10 mice per group) used in this study. Mean weights of 31.33 ± 1.47 g and 28.19 ± 1.10 g were observed in the HFD-fed mice (*n* = 50) and the normal pellet diet (NFD)-fed mice (*n* = 10), respectively. All experimental mice were treated according to the ethical standards of animal experimentation approved by Daegu Haany University Laboratory Animal Ethics Committee (Approval No.: DHU2022-010; Approved on 24 January 2022).

The experimental animals were divided into the following experimental groups (*n* = 10 mice per group):

1. NFD control = 10 mL/kg of vehicle (distilled water)-administered mice with NFD supply
2. HFD control = 10 mL/kg of vehicle (distilled water)-administered mice with HFD supply
3. MET_250_ = 250 mg/kg of metformin-administered mice with HFD supply
4. CBP_400_ = 400 mg/kg of CBP extract-administered mice with HFD supply
5. CBP_200_ = 200 mg/kg of CBP extract-administered mice with HFD supply
6. CBP_100_ = 100 mg/kg of CBP extract-administered mice with HFD supply.

### 2.5. Test Substance Dosage and Administration

The concentrations of CBP extracts chosen for oral dosage—400, 200, and 100 mg/kg—were determined based on dosages reported in our previous studies [12,25,28], and the calculated clinical application dosage (0.5–1 g/head). This calculation considered the differences in body surface volume between humans and mice [29]. CBP extract dosages were provided to mice in a volume of 10 mL/kg following the guidelines of the Korea Food and Drug Administration (KFDA) [30], which is the normal oral administration volume for mice. Additionally, 250 mg/kg of metformin was used as a standard control drug [9,10,11]. The prepared test dosages were orally administered once daily for a period of 84 days. The HFD and NFD control mice were fed a 45% Kcal HFD and a normal pellet diet, respectively (Table S1).

### 2.6. Observation Items

Changes in body weight, weight gains, average feed intake, and the weight of each organ (pancreas, liver, and kidney) were estimated using an automated balance (Precisa, Dietikon, Switzerland), as demonstrated in our previous studies [9,10,11,12,13]. Accumulated fats in the whole body, abdomen, peri-ovarian area, and the abdominal wall area were assessed. Whole blood was collected from the caudal vena cava and separated for biochemical analyses. Blood glucose levels were measured using a blood sugar analyzer (Fuji Medical System Co., LTD., Tokyo, Japan). Blood insulin levels were estimated using a mouse insulin ELISA kit (Alpco Diagnostics, Windham, NH, USA). Glycated hemoglobin (HbA1c) content was calculated by an automated HbA1c measuring system (Infopia, Anyang, Republic of Korea).

For hepatoprotective effects, serum aminotransferase (AST), alkaline phosphatase (ALP), alanine transaminase (ALT), lactate dehydrogenase (LDH), and gamma-glutamyl transferase (GGT) content were measured using an electric blood analyzer (Dri-chem, Prague, Czech Republic). Changes in total cholesterol (TC), TG, low-density lipoprotein (LDL), and high-density lipoprotein (HDL) were measured by an electric blood analyzer (Dri-chem). To measure the fecal lipid content, stool TG and TC content in the extracted fats were analyzed using a total glyceride colorimetric assay kit (Cayman, Ann Arbor, MI, USA) and a total cholesterol assay kit (Cell Bio labs, San Diego, CA, USA), respectively. For nephroprotective effects, blood urea nitrogen (BUN) and creatinine content were measured using an electric blood analyzer (Dri-chem). For histopathological analyses, organ tissues of pancreases, kidneys, and livers were fixed in 10% formalin, stained with hematoxylin and eosin (H&E; Sigma-Aldrich, St. Louis, MO, USA), and the degree of damage to the respective tissue was measured using image analysis software—iSolution FL ver. 9.1 (IMT i-solution Inc., Vancouver, QC, Canada), as explained in our previous reports [9,10,11,25].

The antioxidant properties were analyzed by measuring the Malondialdehyde (MDA) content through lipid peroxidation analysis of the liver tissue [31]. The glutathione (GSH) content was estimated by the 2-nitrobenzoic acid method [32]. Catalase (CAT) and SOD activities were measured by the method of Bolzán et al. [33] and formazan dye method [34], respectively. Glucose metabolism-related liver enzymes (glucokinase—GK, glucose-6-phosphatase—G6pase), and phosphoenolpyruvate carboxykinase (PEPCK) activities were calculated by the methods described in our previous studies [9,10,11].

The mRNA expressions of genes related to fat metabolism (hepatic acetyl-CoA carboxylase 1—ACC1), AMP-activated protein kinases (AMPKα2 and AMPKα1), and adipose tissue adiponectin, uncoupling protein 2 (UCP2), leptin, the binding protein for sterol regulator element (SREBP1c), liver development markers (C/EBPβ and C/EBPα), receptors for cell death (FAS), and peroxisome proliferator (PPARγ and PPARα) were analyzed by real-time quantitative PCR (Bio-Rad, Hercules, CA, USA), as reported in our earlier studies [10,11,25]. The mRNA expressions of glyceraldehyde-3-phosphate dehydrogenase (GAPDH) were used as a standard control to compare the obtained mRNA expressions, and the expressions were calculated following the comparative CT method of Schmittgen and Livak [35]. A list of oligonucleotides used for analyzing the gene expressions is provided in Table S2.

For further details on each experimental method, refer to our previous reports [9,10,11]. In this experiment, the diabetes protective effects of the CBP extracts (400–100 mg/kg) were compared with the 250 mg/kg metformin-administered group [9,10,11,36,37]; this agent is used as a treatment for type 2 diabetes and obesity through representative AMPK activity.

### 2.7. Statistical Analysis

The provided data represent the means ± standard deviation (S.D.) of ten animals. Significance between study groups was assessed using the Levene test, the least-significant differences (LSD) multi-comparison test, one-way analysis of variance (ANOVA), and the Kruskal–Wallis H test, as detailed in our previous studies [9,10,11]. If no significant variance was observed after the Levene test, one-way ANOVA and the LSD multi-comparison test were applied. In cases where significance in variance was observed, verification was conducted using the Kruskal–Wallis H test. Statistical analyses were performed using SPSS ver. 18 (SPSS Inc., Chicago, IL, USA), and differences were deemed statistically significant at *p* < 0.01 or *p* < 0.05.

## 3. Results

### 3.1. Content of Chlorogenic Acid in the CBP Extracts

The HPLC analysis of the CBP extracts detected 1.35 mg/g of chlorogenic acid, calculated according to the standard chlorogenic acid concentration comparison (Figure S1).

### 3.2. Evaluation of In Vitro Antioxidant Activity

**Figure 1:**
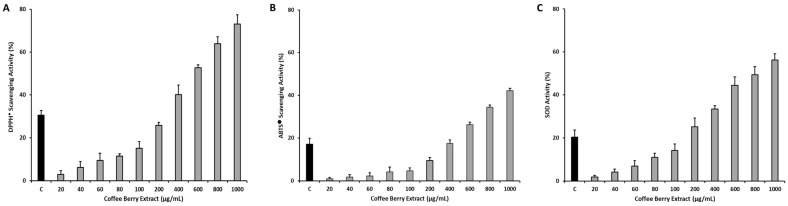
Antioxidant activity of CBP extracts. (**A**) DPPH-radical scavenging activity, (**B**) ABTS-radical scavenging activity, and (**C**) SOD activity of CBP extracts at different concentrations ranging from 0 to 1000 µg/mL; C: Control (L-ascorbic acid was used as a control treatment); CBP: Coffee berry pulp extracts.

The findings of the analysis of antioxidant activity are presented in Figure 1. Notably, the CBP extracts exhibited a significant (*p* < 0.05) and concentration-dependent increase in all examined antioxidants, with the highest antioxidant activity observed at the maximum tested concentration of 1000 µg/mL. Specifically, the DPPH^●^ scavenging activity assay, ABTS^●^ scavenging activity assay, and SOD activity assay demonstrated the highest antioxidant activity levels of 73.10 ± 4.27%, 41.18 ± 1.14%, and 56.24 ± 2.81%, respectively. Conversely, the lowest levels of antioxidant activity observed with the DPPH^●^ scavenging activity assay, ABTS^●^ scavenging activity assay, and SOD activity assay were 2.99 ± 1.69%, 0.93 ± 0.74%, and 1.89 ± 0.70%, respectively. In comparison, the control (L-ascorbic acid) exhibited a DPPH^●^ scavenging activity of 30.46 ± 2.21%, ABTS^●^ scavenging activity of 17.08 ± 2.78%, and SOD activity of 20.38 ± 3.22%. Furthermore, it is noteworthy that the CBP extracts demonstrated a consistent trend in antioxidant activity across all tested antioxidant assays, and these outcomes were statistically significant (*p* < 0.05) when compared to the control treatment.

### 3.3. Anti-Obesity Properties

#### 3.3.1. Effects on Body Weights and Average Feed Intake

**Figure 2:**
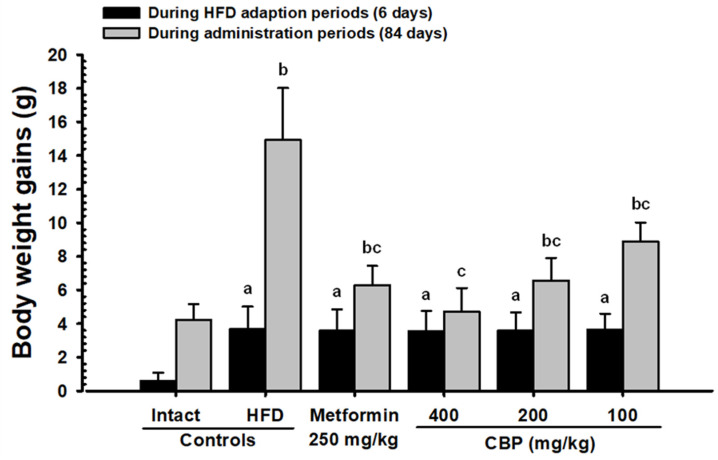
Body weight gains in mice supplied with either NFD or HFD. Values are expressed as means ± S.D. of 10 mice; NFD: Normal pellet diet; HFD: 45% Kcal high-fat diet; CBP: Coffee berry pulp extracts; THSD: Tukey’s Honest Significant Difference; DT3: Dunnett’s T3; NFD control: Vehicle (10 mL/kg distilled water) orally administered mice with NFD supply; HFD control: Vehicle (10 mL/kg distilled water) orally administered mice with HFD supply; ^a^ *p* < 0.01 as compared with NFD control by THSD test; ^b^ *p* < 0.01 as compared with NFD control by DT3 test; ^c^ *p* < 0.01 as compared with the HFD control using the DT3 test.

After six days of HFD supply, significant increases in body weights (*p* < 0.01) were noted in the HFD control group compared to the NFD control group. The changes in body weight gains during 84 days of experimental substance administration and a one-week HFD adaptation period also increased significantly compared to the NFD control group. Meanwhile, significant weight losses in the CBP extract-administered groups CBP_400_, CBP_200_, and CBP_100_, from 21, 28, and 42 days after the start of administration, respectively, and in the MET_250_ group, 28 days after the first administration, were noted (*p* < 0.01; *p* < 0.05). All experimental groups, including the MET_250_ group, showed significant (*p* < 0.01) decreases in body weight gain during the 84-day experimental period compared to the HFD control group. Additionally, the observed inhibitory effect on weight gain due to the HFD supply in the CBP_200_ group were comparable to those observed in the MET_250_ group (Figure 2 and Figure S2; Table S3). Significant (*p* < 0.01) decreases in average feed intake were observed in the HFD control group compared to the NFD control. However, these changes were not significant in all tested dosage groups, including CBP_400_ (Table S3).

#### 3.3.2. Effects on Abdominal and Body Fat Volume

The HFD control group showed a significant (*p* < 0.01) increase in accumulated fats in the abdominals and body, compared to the NFD control. By contrast, fat accumulation in the abdominals and body of mice supplied with all CBP extract doses (CBP_400_, CBP_200_, and CBP_100_) was significantly (*p* < 0.01) reduced. In particular, the inhibitory effect of HFD-induced increases in fat accumulation in the CBP_200_ group was comparable to the MET_250_ group (Figures S3 and S4).

#### 3.3.3. Change in Fat Weight

The HFD control group showed a significant (*p* < 0.01) increase in the relative and absolute weight of ovarian periphery and abdominal wall accumulated fats as compared to the NFD control. However, significant (*p* < 0.01; *p* < 0.05) reductions observed in ovarian peripheral and abdominal wall accumulated fat in all CBP extract-administered groups were in a dose-dependent manner. Inhibitory effects on the HFD-induced increase in the absolute and relative weight of ovary and abdominal wall accumulated fat in the CBP_200_ group were comparable to the MET_250_ group (Figure S4; Table 1 and Table 2).

#### 3.3.4. Histopathological Observations for Accumulated Fats around the Abdominal Wall and the Ovaries

The HFD control group showed a significant (*p* < 0.01) enlargement of fat cells and a significant (*p* < 0.01) increase in the thickness and diameter of fat tissue accumulated around the abdominal wall and the ovaries, as compared to the NFD control. However, significant (*p* < 0.01) reduction in adipose tissue thickness and adipocyte diameter in the ovary-peripheral fat and fat accumulated around the abdominal wall were observed in all the CBP extract-administrated groups in a dose-dependent manner. In particular, the inhibitory effect on the HFD-induced increase in the thickness and diameter of fat cells in the adipose tissue around the ovary and abdominal wall was noted in the CBP_200_ group, comparable to those observed in the MET_250_ group (Figure S5; Table 3).

#### 3.3.5. Histopathological Observations for Pancreatic Exocrine Zymogen Granules

**Figure 3:**
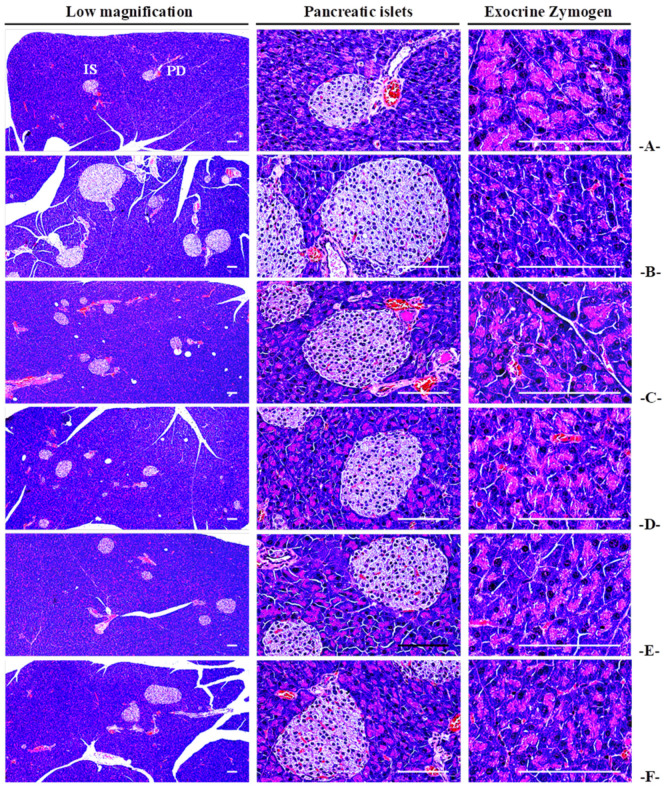
Representative general histological images of the pancreas, taken from mice supplied with either NFD or HFD. (**A**) Vehicle (10 mL/kg distilled water) orally administered mice with NFD supply (NFD control); (**B**) Vehicle (10 mL/kg distilled water) orally administered mice with HFD supply (HFD control); (**C**) Metformin (250 mg/kg) orally administered mice with HFD supply (MET_250_); (**D**) CBP (400 mg/kg) orally administered mice with HFD supply (CBP_400_); (**E**) CBP (200 mg/kg) orally administered mice with HFD supply (CBP_200_); (**F**) CBP (100 mg/kg) orally administered mice with HFD supply (CBP_100_); NFD: Normal pellet diet; HFD: 45% Kcal high-fat diet; CBP: Coffee berry pulp extracts; IS: Pancreatic islet; PD: Pancreatic secretory duct; All hematoxylin and eosin stained; Scale bars: 80 µm.

The HFD control group showed a significant (*p* < 0.01) decrease in the percentage of pancreatic exocrine zymogen granules compared to the NFD control. However, significant (*p* < 0.01) increase in the rates of zymogen granules were noted in all CBP extract-administered groups in a dose-dependent manner. The inhibitory effects of HFD-induced reduction in the ratio of zymogen granules in the CBP_200_ group were comparable to the MET_250_ group (Figure 3; Table S4).

### 3.4. Anti-Diabetic Properties

#### 3.4.1. Effects on Insulin Content, Blood Sugar, and HbA1c Ratio

**Figure 4:**
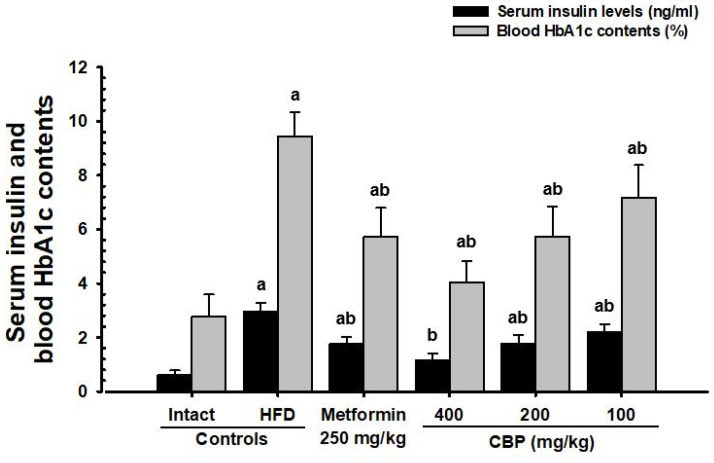
Serum insulin and blood HbA1c content in mice supplied with either NFD or HFD. Values are expressed as mean ± S.D. of 10 mice; NFD: Normal pellet diet; HFD: 45% Kcal high-fat diet; CBP: Coffee berry pulp extracts; HbA1c: Glycated hemoglobin—hemoglobin A1c; THSD: Tukey’s Honest Significant Difference; NFD control: Vehicle (10 mL/kg distilled water) orally administered mice with NFD supply; HFD control: Vehicle (10 mL/kg distilled water) orally administered mice with HFD supply; ^a^ *p* < 0.01 as compared with the NFD control using the THSD test; ^b^ *p* < 0.01 as compared with the HFD control using the THSD test.

The HFD control group showed significant (*p* < 0.01) increases in blood insulin content, blood sugar, and HbA1c ratio compared to the NFD control. However, these changes were significantly (*p* < 0.01) and dose-dependently reduced in all groups administered with the CBP extracts (400–100 mg/kg). The inhibitory effects of HFD-induced increases in blood insulin, blood glucose, and HbA1c ratio were confirmed in the CBP_200_ group, as comparable to the MET_250_ group (Figure 4; Table 4).

#### 3.4.2. Effects on Pancreatic Weight

A significant reduction in pancreatic relative weight was noted in the HFD control group compared to the NFD control. However, a significant (*p* < 0.01; *p* < 0.05) and dose-dependent increase in pancreatic relative weight was observed in all CBP extract-supplied groups. In particular, the inhibitory effects on the HFD-induced reductions in the pancreatic relative weight in the CBP_200_ group were comparable to the MET_250_ group. By contrast, no notable change in pancreatic absolute weight was found in the HFD control, and no significant change in pancreatic absolute weight in all tested groups, including the CBP_200_, was found compared to the HFD control group (Table 1 and Table 2).

#### 3.4.3. Effects on the General Histopathology of Pancreatic Islands

The HFD control group showed a significant (*p* < 0.01) proliferation of pancreatic islands and significant (*p* < 0.01) increases in the number and average diameter of pancreatic islands, compared to the NFD control group. However, the reduction in the diameter and number of pancreatic islands in all CBP extract-supplied groups was significant (*p* < 0.01) and in a dose-dependent manner. In particular, the inhibitory effects of HFD-induced increases in the diameter and number of pancreatic islands in the CBP_200_ group were compared to the MET_250_ group (Figure 3; Table S4).

#### 3.4.4. Effects on the Immunohistochemistry of Pancreatic Islands

**Figure 5:**
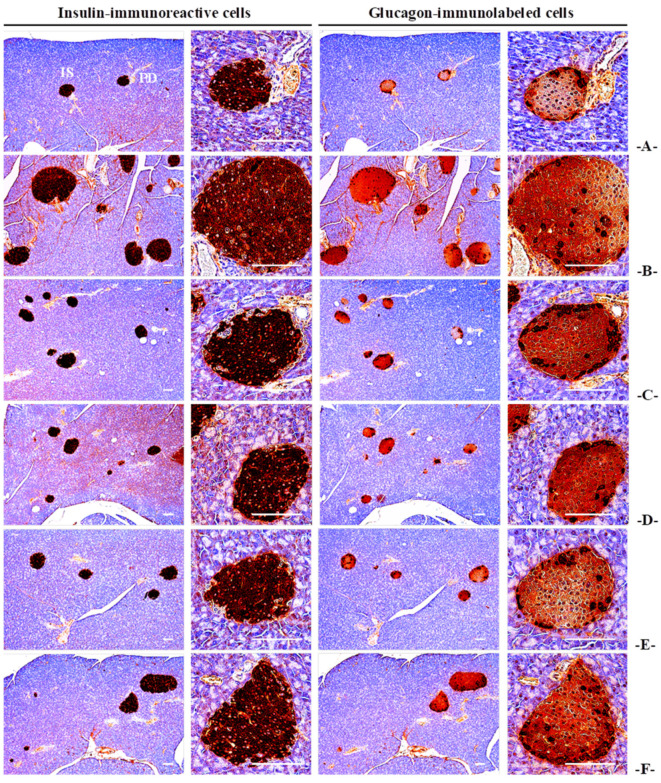
Representative histological images of the insulin- and glucagon-immunoreactive cells in the pancreas, taken from mice supplied with either NFD or HFD. (**A**): Vehicle (10 mL/kg distilled water) orally administered mice with NFD supply (NFD control); (**B**): Vehicle (10 mL/kg distilled water) orally administered mice with HFD supply (HFD control); (**C**): Metformin (250 mg/kg) orally administered mice with HFD supply (MET_250_); (**D**): CBP (400 mg/kg) orally administered mice with HFD supply (CBP_400_); (**E**): CBP (200 mg/kg) orally administered mice with HFD supply (CBP_200_); (**F**): CBP (100 mg/kg) orally administered mice with HFD supply (CBP_100_); NFD: Normal pellet diet; HFD: 45% Kcal high-fat diet; CBP: Coffee berry pulp extracts; IS: Pancreatic islet; PD: Pancreatic secretory duct; All samples were immunostained by avidin–biotin–peroxidase complex; Scale bars: 80 µm.

The HFD control group showed a significant (*p* < 0.01) increase in the number of immune-responsive cells for insulin and glucagon and an increase in the insulin/glucagon cell ratio compared to the NFD control. However, these changes were significantly (*p* < 0.01) and dose-dependently decreased in all CBP extract-supplied groups. In the CBP_200_ group, the inhibitory effects of HFD-induced increases in the number of immune-responsive cells and the ratio of insulin/glucagon cells were observed compared to the MET_250_ group (Figure 5; Table S4).

### 3.5. Effects on Hyperlipidemia

#### 3.5.1. Effects on Blood TC, TG, and LDL Content

In the HFD control group, a significant (*p* < 0.01) increase in the TC, TG, and LDL content was observed compared to the NFD control. However, significant (*p* < 0.01; *p* < 0.05) reductions in the TC, TG, and LDL content in the blood observed in all CBP extract-administered groups were in a dose-dependent manner. In particular, the inhibitory effects of HFD-induced increase in the blood TG, TC, and LDL content were confirmed in the CBP_200_ group as compared to the MET_250_ group (Table 4).

#### 3.5.2. Effects on HDL Content in Blood

The HFD control group showed a significant (*p* < 0.01) decrease in the HDL content in blood compared to the NFD control. However, the decrease in HDL content was significantly (*p* < 0.01) and dose-dependently increased in groups supplied with the CBP extracts (400–100 mg/kg). In particular, the inhibitory effects of HFD-induced reduction in blood in the CBP_200_ group were comparable to the MET_250_ group (Table 4).

#### 3.5.3. Effects on Fecal Lipid Content

**Figure 6:**
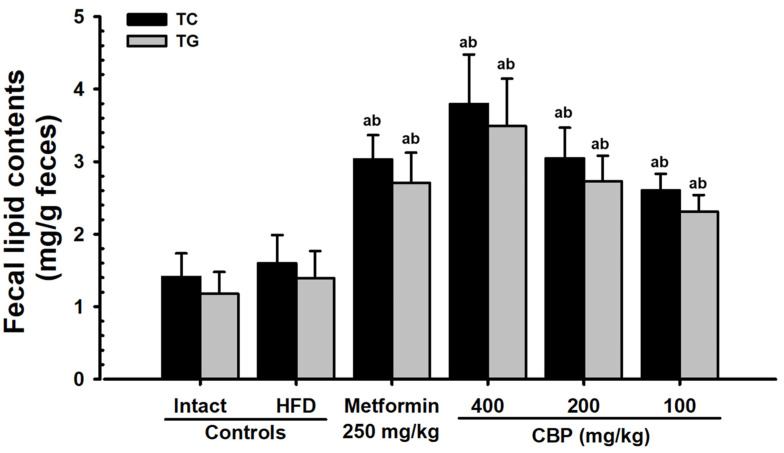
Fecal TC and TG content in mice supplied with either NFD or HFD. Values are expressed as mean ± S.D. of 10 mice; NFD: Normal pellet diet; HFD: 45% Kcal high-fat diet; CBP: Coffee berry pulp extracts; TC: Total cholesterol; TG: Triglyceride; THSD: Tukey’s Honest Significant Difference; NFD control: Vehicle (10 mL/kg distilled water) orally administered mice with NFD supply; HFD control: Vehicle (10 mL/kg distilled water) orally administered mice with HFD supply; ^a^ *p* < 0.01 as compared with the NFD control using the THSD test; ^b^ *p* < 0.01 as compared with the HFD control using the THSD test.

The minor increases in the TG and TC content in fecal lipids in the HFD control group were not significant compared to the NFD control. However, all three doses of the CBP extract administration significantly (*p* < 0.01) and dose-dependently increased the fecal TG and TC content. In particular, the inhibitory effects of HFD-induced increase in the fecal TG and TC content were confirmed in the CBP_200_ group comparable to the MET_250_ group (Figure 6).

### 3.6. Liver Damage

#### 3.6.1. Liver Weight

In the HFD control group, the absolute liver weight increased significantly compared to the NFD control. However, significant (*p* < 0.01) and dose-dependent reductions in absolute liver weight were observed in all CBP extract-administered groups. In particular, the inhibitory effects of HFD-induced increase in absolute liver weight in the CBP_200_ group were confirmed as comparable to the MET_250_ group. On the contrary, no significant changes in the relative weight of the liver were found in the HFD control group, and no notable change in the relative weight of the liver was noted in all tested groups, including the CBP_100_, as compared to the HFD control (Table 1 and Table 2).

#### 3.6.2. Changes in Serum ALT, AST, ALP, GGT, and LDH Content

In the HFD control group, significant (*p* < 0.01) increases in blood ALT, AST, ALP, GGT, and LDH content were observed compared to the NFD control. However, these changes were significantly (*p* < 0.01) and dose-dependently reduced in groups supplied with the CBP extracts (400–100 mg/kg). In particular, the inhibitory effects of HFD-induced increase in blood ALT, AST, ALP, GGT, and LDH content in the CBP_200_ group was comparable to the MET_250_ group (Table 5).

#### 3.6.3. Effects on Histopathology of Hepatic Fat Change Rate and Hepatocellular Diameter

The HFD control group showed fatty liver findings characterized by hepatocellular hypertrophy due to significant intrahepatic fat accumulation, and significant (*p* < 0.01) increases in the hepatic fat change rate and hepatocellular diameter were confirmed compared to the NFD control. However, in all the CBP extract-administered groups, the decreases in the rate of change in liver fat and average hepatocellular diameter were significant (*p* < 0.01) and in a dose-dependent manner. In particular, the inhibitory effects of HFD-induced increases in the change rate of hepatic fat and hepatocellular diameter in the CBP_200_ group were confirmed as comparable to the MET_250_ group (Figure S6; Table 6).

### 3.7. Kidney Damage

#### 3.7.1. Effects on Kidney Weight

In the HFD control group, the absolute weight of the kidneys significantly (*p* < 0.01) increased as compared to the NFD control. However, a significant (*p* < 0.01) reduction in kidney absolute weight was observed in all CBP extract-administered groups. In particular, the inhibitory effects of HFD-induced increase in the absolute weight of the kidneys in the CBP_200_ group were confirmed as comparable to the MET_250_ group. On the contrary, no significant changes in relative weights of the kidneys were found in the HFD control group compared to the NFD control, and no noticeable change in relative weights of the kidneys was found in all experimental groups, including the MET_250_ group, compared to the HFD control (Table 1 and Table 2).

#### 3.7.2. Effects on Blood BUN and Creatinine Content

The HFD control group showed significant (*p* < 0.01) increases in BUN and creatinine content in the blood, compared to the NFD control. However, these changes were significantly (*p* < 0.01) and dose-dependently reduced in groups administered with the CBP extracts (400–100 mg/kg). In particular, the inhibitory effects of HFD-induced increases in the blood creatinine content and BUN were confirmed in the CBP_200_ group, comparable to the MET_250_ group (Table 5).

#### 3.7.3. Histopathology of Kidney

The HFD control group showed a significant (*p* < 0.01) increase in the number of denatured tubules, characterized by degenerative vacuolated renal tubes, as compared to the NFD control. However, the number of denatured tubules was significantly (*p* < 0.01) and dose-dependently reduced in all CBP extract-supplied groups. In particular, the inhibitory effects on the HFD-induced increases in the renal tubule vacuolation were confirmed in the CBP_200_ group as comparable to the MET_250_ group (Figure S7; Table 6).

### 3.8. Effect on the Hepatic Antioxidant Defense Systems

#### 3.8.1. Lipid Peroxidation

The HFD control group showed a significant (*p* < 0.01) increase in MDA content as compared to the NFD control. However, a significant (*p* < 0.01) and dose-dependent reduction in liver lipid peroxidation was found in all CBP extract-administered groups. Inhibiting effects on the HFD-induced increases in lipid peroxidation in the CBP_200_ group was comparable to the MET_250_ group (Table 7).

#### 3.8.2. Changes in Hepatic SOD, CAT, and GSH Activity

The HFD control group showed significant (*p* < 0.01) decreases in SOD, CAT, and GSH activity in liver tissue, which are endogenous antioxidant enzymes, compared to the NFD control. However, these changes were significantly (*p* < 0.01; *p* < 0.05) and dose-dependently reversed in the groups supplied with the CBP extracts (400–100 mg/kg). In particular, the inhibitory effect on the HFD-induced decrease in the hepatic SOD, CAT, and GSH activity confirmed in the CBP_200_ group was comparable to the MET_250_ group (Table 7).

### 3.9. Sugar Metabolism-Related Enzyme Activity

#### 3.9.1. Effects on Hepatic GK Activity

The HFD control group showed a significant (*p* < 0.01) decrease in GK activity, which is a glycolysis enzyme in the liver tissue, compared to the NFD control. However, significant (*p* < 0.01; *p* < 0.05) and dose-dependent increase in the GK activity was noted in all CBP extract-administered groups. In particular, the inhibitory effect on the HFD-induced decrease in the GK activity confirmed in the CBP_200_ group was comparable to the MET_250_ group (Table 8).

#### 3.9.2. Changes in Liver PEPCK and G6phase Activity

The HFD control group showed significant (*p* < 0.01) increases in the liver glycosynthetic enzyme (G6phase) and glycolytic enzyme (PEPCK) activity, compared to the NFD control. However, the increases in PEPCK and G6phase activity were significantly (*p* < 0.01) and dose-dependently reduced in the groups supplied with the CBP extracts (400–100 mg/kg). In particular, the inhibiting effect on the HFD-induced increase in the G6phase and PEPCK activity confirmed in the CBP_200_ group was comparable to the MET_250_ group (Table 8).

### 3.10. Changes in Lipid Metabolism-Related Gene Expressions

#### 3.10.1. Changes in Gene Expressions in Liver Tissue

The HFD control group showed significant (*p* < 0.01) reductions in liver AMPKα1 and AMPKα2 mRNA expressions, along with an increase in ACC1 mRNA expression, compared to the NFD control. However, these changes in mRNA expression were significantly (*p* < 0.01) reversed in groups supplied with the CBP extracts (400–100 mg/kg). In particular, the inhibiting effect on the HFD-induced increase in the ACC1, AMPKα1, and AMPKα2 mRNA expressions confirmed in the CBP_200_ group was comparable to the MET_250_ group (Table 9).

#### 3.10.2. Changes in Gene Expressions in Adipose Tissue

The HFD control group showed significant (*p* < 0.01) decreases in the PPARα, adiponectin, and UCP2 mRNA expressions with significantly increased mRNA expressions of C/EBPγ, C/EBPα, leptin, SREBP1c, FAS, and PPAR in adipose tissue as compared to the NFD control. However, these mRNA expressions were significantly (*p* < 0.01; *p* < 0.05) reversed in all the groups supplied with CBP extracts (400–100 mg/kg). In particular, the inhibitory effects on the HFD-induced increases in C/EBPγ, C/EBPα, leptin, SREBP1c, FAS, and PPAR mRNA expression and decreases in PPARα, adiponectin, and UCP2 mRNA expression confirmed in the CBP_200_ group were comparable to the MET_250_ group (Table 10).

## 4. Discussion

Reactive oxygen species (ROS) generate activated oxygen species, such as superoxide anion radical (O_2_^●−^), singlet oxygen (O_2_), hydrogen peroxide (H_2_O_2_), and hydroxyl radical (^●^OH) [38,39,40]. Excessive ROS production has been implicated in numerous diseases [41,42], leading to various detrimental effects, including cellular necrosis, enzyme inactivation, apoptosis, DNA damage, and lipid peroxidation. These effects can contribute to the development of obesity, cardiovascular diseases, inflammation, neural diseases, cancer, and aging [41,42]. One well-established mechanism through which antioxidants inhibit lipid peroxidation is by scavenging free radicals [43]. Antioxidants play a critical role in shielding cells from the harmful consequences of ROS and are widely used in the fields of medicine and food production. The quest for natural antioxidants from sources such as seaweed, microorganisms, and plants has gained prominence in recent years [38,41,44].

The antioxidant activity of CBR extracts was evaluated using in vitro assays, including the DPPH^●^ and ABTS^●^ scavenging activity assays, and the SOD activity assay. DPPH is a stable free radical characterized by its absorbance at 517 nm, which decreases significantly in the presence of radical scavengers that provide hydrogen atoms or electrons, resulting in a stable diamagnetic molecule [45]. Previous research, including studies by Geremu et al. [46], Chen et al. [47], and Eswari et al. [48], has reported substantial DPPH^●^ scavenging activity in coffee cherry pulp extracts. Consistent with these findings, the DPPH^●^ scavenging activity of the CBR extracts revealed concentration-dependent increases, with the highest DPPH^●^ scavenging activity of 73.10 ± 4.27% observed at 1000 µg/mL CBP extracts, significantly (*p* < 0.05) different from the control treatment (L-ascorbic acid: 30.46 ± 2.21%). Similarly, the ABTS^●^ scavenging assay of the CBR extracts showed concentration-dependent increases, with the highest ABTS^●^ scavenging activity of 42.18 ± 1.14% recorded at 1000 µg/mL CBP extract, significantly (*p* < 0.05) different from the control treatment (L-ascorbic acid: 17.08 ± 2.78%).

SOD is a primary antioxidant enzyme that counteracts the toxic effects of O_2_^−^ radicals by catalyzing the conversion of H_2_O_2_ and O_2_ [49]. Imbalances between free radical production and antioxidant levels can lead to oxidative stress, characterized by reduced SOD enzyme activity [50]. Enhancing SOD activity can help mitigate oxidative stress, and its inhibition is considered crucial for managing diabetes and related complications [51,52]. Saewan [53] reported an SOD activity of 74.53 ± 0.92% in crude coffee berry extracts. In our study, the CBR extracts exhibited concentration-dependent increases in SOD activity, with the highest SOD activity of 56.24 ± 2.81% observed at 1000 µg/mL, significantly (*p* < 0.05) different from the control treatment (L-ascorbic acid: 20.38 ± 3.22%). Elevated SOD activity in the CBP extracts holds potential for reducing the adverse effects of free radicals, thus aiding in the prevention of degenerative and chronic diseases.

It is important to note that antioxidant capacity can vary when assessed through different in vitro assays [54,55,56,57,58]. Pellegrini et al. [57] observed variations in the rankings of antioxidant capacities for various fruits, vegetables, and beverages when different assays, including DPPH^●^ and ABTS^●^ radical scavenging activity assays, were employed. This underscores the need for caution when interpreting antioxidant capacity data derived from different assays [59]. Similarly, in our study, the antioxidant activity of CBR extracts analyzed using DPPH^●^ and ABTS^●^ scavenging assays and the SOD activity assay yielded significantly different results. However, a consistent pattern of concentration-dependent increase in antioxidant activity across all tested antioxidant assays was observed. Higher DPPH^●^ and ABTS^●^ scavenging activity can be attributed to the presence of phenolic compounds. Nevertheless, further investigations are warranted to elucidate the factors influencing the antioxidant activity of the BCP extracts. Consequently, we sought to explore whether the CBP extracts possess protective properties against obesity induced by HFD in mild type 2 diabetic and obese ICR mice.

In rodents, HFD feeding causes obesity, hyperglycemia, fatty liver, insulin resistance, hyperlipidemia, and renal degeneration [9,10,11]. Therefore, the current HFD-fed rodent model is considered one of the most frequently employed experimental animal models for the development of drugs for diabetes, obesity, and related complications [9,10,11,60,61]. In this study, the HFD control group showed significant increases in body weight compared to the NFD control group six days after HFD supply. Meanwhile, the CBP_400_, CBP_200_, CBP_100_, and MET_250_ groups showed significant weight loss after 21, 28, 42, and 28 days, respectively, after the first administration. The significant decreases in weight gains during the 84-day test material supply period were also confirmed in all tested groups, including the MET_250_. In addition, the inhibitory effects of HFD-induced body weight and weight gain in the CBP_200_ group were comparable to that of the MET_250_ group; thus, CBP extract administration provides clear evidence showing excellent inhibitory effects on HFD-induced weight gain in a dose-dependent manner.

All tested concentrations of the CBP extracts demonstrated a significant accumulation of fats, confirming dose-dependent inhibitory effects on adipocyte hypertrophy. Notably, the inhibitory effect on the HFD-induced adipocyte hypertrophy and fat accumulation observed in the CBP extract (200 mg/kg)-administered group was comparable to the MET_250_ group. Therefore, it is considered clear evidence of a dose-dependent improvement in obesity using CBP extracts in HFD-fed mice. Although the weight loss in mice receiving CBP extracts may be attributed to a decrease in high-fat feed intake, considering the calorie intake of NFD (4.00 kcal/g) and HFD (4.73 kcal/g), it is believed that this weight loss is not merely a result of reduced feed intake. A similar reduction in high-fat feed intake has been reported in earlier studies on anti-obesity and anti-type 2 diabetes functional food materials [62,63].

Histopathologically, significant reductions in accumulated zymogen granules were observed in the HFD control compared to the NFD control, indicating the promotion of fat digestion-related pancreatic enzymes’ secretion. Conversely, the experimental groups treated with CBP extracts (400–100 mg/kg) showed significant and dose-dependent suppression of the proportion of pancreatic zymogen granules. Particularly, the inhibitory effects on HFD-induced reductions in the proportion of pancreatic zymogen granules were confirmed in the CBP_200_ group, comparable to the MET_250_ group. Thus, it is considered convincing evidence that CBP extract administration improves obesity by inhibiting fat absorption, mediated through the regulating of fat-digesting enzyme secretion in the pancreases.

HbA1c is a crucial clinical indicator for determining long-term hyperglycemia [64,65], as insulin resistance typically leads to increased HbA1c and insulin content [66]. Long-term HFD supply resulted in histopathological increases and expansion of pancreatic islets, glucagon, and insulin-producing cells, and insulin/glucagon cell ratio to maintain blood sugar homeostasis [67,68]. In this study, significant increases in blood HbA1c, glucose, and insulin, along with increases in the number and expansion of pancreatic islets and increases in glucagon, insulin, and insulin/glucagon cell ratio were histopathologically confirmed, leading to typical insulin-resistant type 2 diabetes. The CBP extract (400–100 mg/kg)-administered groups significantly suppressed blood glucose, HbA1c, and insulin content, along with immunohistochemical and histological deviations in the endocrine part of the pancreas in a dose-dependent manner. In particular, the inhibitory effects on HFD-induced suppression of insulin-resistant type 2 diabetes in the CBP_200_ group were comparable to the MET_250_ group, making it clear evidence that CBP extract administration shows a blood glucose improvement effect through pancreatic endocrine function in a dose-dependent manner.

Reduced HDL content along with increases in TG, TC, and LDL content are usually observed in hyperlipidemia caused by high-calorie intake [69]. In this experiment, all three doses of CBP extracts significantly reduced blood TG, TC, and LDL content and increased blood HDL content. These results were similar to previous studies on the antihyperlipidemic effects of candidate substances showing increased HDL content along with decreased blood TG, TC, and LDL content [63,70]. Particularly, the inhibitory effect on HFD-induced hyperlipidemia in the CBP_200_ group was comparable to the MET_250_ group, judging it as clear evidence of the suppressing effects of HFD-induced hyperlipidemia by CBP extract administration. Furthermore, the hyperlipidemia improvement effects by CBP extracts were accompanied by increases in fecal TC and TG content, comparable to the MET_250_ group, and histopathological increases in exocrine pancreatic zymogen content were confirmed. Thus, it is believed that this is due to the increase in lipid excretion resulting from the suppression of lipid digestion and the decrease in absorption through the regulation of secretion of digestive enzymes in the pancreas.

Fats’ degradation and accumulation in hepatocytes enhances blood ALT, AST, ALP, GGT, and LDH content [71], making them the most common indicators of blood chemistry to identify liver damage [72]. The HFD-induced increases in absolute liver weights and the increases in blood ALT, AST, ALP, GGT, and LDH content observed in all the CBP extract-administered groups were dose-dependent. Additionally, histopathological examination showed that CBP extract administration significantly suppressed liver fat changes and consequent hypertrophy of hepatocytes. These findings are considered clear evidence of HFD-induced NAFLD amelioration effects at a CBP extract dose of 200 mg/kg compared to the 250 mg/kg of metformin. These results align with previous reports using HFD-induced mouse models [70,71,73].

Creatinine and BUN content in blood are the most representative blood chemical indicators of kidney status [50]. The HFD control mice showed significant increases in absolute kidney weights and increases in creatinine and BUN content. Furthermore, histopathological findings of tubular vacuolization characterized by fat droplet infiltration were confirmed. However, the development of diabetic nephropathy was significantly and dose-dependently suppressed by all three doses of CBP extracts. Similar to the study of Chi et al. [74], reductions in creatinine and BUN content were confirmed in this study, indicating an inhibitory effect on diabetic nephropathy. In particular, the improvement effects on HFD-induced diabetic nephropathy in the CBP_200_ group were comparable to the MET_250_ group. Thus, it is considered clear evidence of the HFD-induced diabetic nephropathy improvement effect by supplying CBP extracts.

Free radicals play a significant role in the induction of diabetes and the related metabolic syndrome [75]. ROS generated by lipid peroxidation cause damage to surrounding tissues [76], increasing lipid peroxidation in major parenchymal organs [9,10,11,25]. The inhibition of CAT and SOD activity, reduction of GSH content, and lipid peroxidation are considered crucial for treating diabetes and its related complications [51,52]. Similar to previous studies [66,77], lipid peroxidation in HFD control mice showed increased MDA content in liver parenchyma and decreased activity of GSH, CAT, and SOD enzymes. The CBP supply (400–100 mg/kg) significantly suppressed lipid peroxidation and the related antioxidant defense systems in a dose-dependent manner. Furthermore, promising antioxidant effects were observed at a CBP extract dose of 200 mg/kg, comparable to that of 250 mg/kg of metformin.

The GK enzyme in the liver regulates blood sugar by facilitating glycogen storage or stimulating the utilization of blood sugar as energy [78,79]. On the other hand, liver PEPCK and G6pase are associated with gluconeogenesis, leading to the release of glucose in the liver and an increase in blood glucose levels [80,81]. Chung et al. [66] reported an increase in PEPCK and G6pase activities with noticeable decreases in GK activity in HFD-fed hyperglycemic controls. Similarly, in this study, there were significant increases in PEPCK and G6pase activities and decreases in GK activity in liver tissue in the HFD control group. However, the administration of CBP extracts (400–100 mg/kg) significantly and dose-dependently suppressed these changes in liver enzymatic activities. In particular, improvement effects of the HFD-induced changes in PEPCK, G6pase, and GK activities in the CBP_200_ group were confirmed to be comparable to the MET_250_ group. This is considered clear evidence that CBP extracts exhibit properties regulating glucose metabolism-related enzymes in a dose-dependent manner.

The mRNA expressions of genes linked with fat metabolism in adipose and liver tissue were evaluated to understand the mode of action of a drug candidate for diabetes and associated syndromes, such as NAFLD. Estimating AMPK activity and expression in adipose and liver tissue is considered one of the most critical factors for the cell signaling pathway, as it regulates blood glucose and fat metabolism by inhibiting glucose and fat synthesis and accelerating fat oxidation. Therefore, observing the mRNA expression pattern of AMPK and proteins linked with the adipose and liver AMPK signal transduction system is of utmost importance [82,83]. The HFD control mice showed decreased expressions of AMPKα1 and AMPKα2 mRNA with an increased expression of ACC1 mRNA in the liver tissue and increased expressions of C/EBPβ, C/EBPα, SREBP1c, FAS, leptin, and PPARγ mRNA with decreased expressions of PPARα, adiponectin, and UCP2 mRNA in the adipose tissue. However, the supply of CBP extracts (400–100 mg/kg) significantly suppressed the HFD-induced changes in the gene expression of lipid metabolism-related genes and AMPK. Noticeable improvement effects in the HFD-induced changes in lipid metabolism-associated genes and AMPK were confirmed in the CBP_200_ group, compared to the MET_250_ group. These results confirm similar findings detected in previous studies [84,85,86]. Therefore, it shows clear evidence for the improvement of lipid metabolism by CBP extract administration through the AMPK-regulated increase in fatty acid oxidation and inhibition of lipid synthesis observed in a dose-dependent manner.

The higher consumption of coffee, estimated at an average of 2.25 billion cups per day worldwide [87], results in the generation of wastewater and various by-products, including coffee peels, parchment, pulp, and spent coffee grounds [87,88]. Notably, among these, coffee pulp stands out as a major underutilized by-product at the farm level during coffee processing. Due to its elevated antioxidant activities [15,16], with the reported anti-obesity characteristics from this study, the sustainable utilization of coffee pulp as a source of polyphenols and pectin presents an opportunity to address the waste generated in coffee processing at the farm level [89]. This could contribute to the development of antioxidant drinks and functional foods for health, derived from coffee cherry pulp. Furthermore, the by-products derived from the coffee production chain can be characterized and repurposed for various applications in agribusiness and other sectors, fostering a sustainable agricultural economy. Potential uses include recycling as fertilizer, harnessing energy, and recovering biomaterials [88].

Coffee is broadly categorized into three major varieties: *C. arabica*, *Coffea robusta*, and *Coffea riberica* [90], with *C. arabica* being the most globally produced. Consequently, we focused our investigation on the antioxidant and anti-obesity effects of *C. Arabica*. A prospective study could further explore the bioactivities of other coffee species, such as *C. robusta* and *C. riberica*. While our current study observed the favorable antioxidant, anti-obesity, and anti-diabetic properties of CBP extracts, using an animal model and oral dosages of 400–100 mg/kg, it is important to note that these specific dosages may not directly correlate to those suitable for humans. Additionally, although the study highlights positive outcomes, it may not comprehensively address potential side effects or adverse reactions related to the extract’s use. Therefore, future studies should focus on investigating these aspects. Dietary polyphenols are well known for their antioxidant and anti-obesity properties [91], providing protection against obesity by inhibiting adipogenesis through the modulation of signaling pathways that regulate anti-inflammatory and antioxidant responses. Cellular and animal studies have indicated that dietary polyphenols offer protection against obesity through the modulation of signaling pathways, regulating anti-inflammatory and antioxidant responses [12,25,28]. Consequently, we hypothesize that the anti-obesity and anti-diabetic properties of CBP extracts, as reported in this study, are linked to their antioxidant characteristics due to the presence of phenolic compounds. However, a future study could delve into the analysis of coffee constituents through NMR spectra of CBP extracts. This analysis could enhance our understanding of the correlation between the elements constituting CBP extracts and their influences on the antioxidant response in animals, as well as on changes in lipid and sugar metabolisms.

## 5. Conclusions

In developing a natural product-based functional food material or a new drug candidate for the improvement of diabetes and related complications, including diabetic nephropathy, NAFLD, and hyperlipidemia, the dose-dependent improvement effects of CBP extracts (400–100 mg/kg) were investigated in a mild type 2 obese diabetic mouse model. The in vitro antioxidant activities of CBP extracts exhibited concentration-dependent improvements across all tested antioxidant assays, which included DPPH^●^ and ABTS^●^ scavenging activity assays, as well as an SOD activity assay. Furthermore, dose-dependent improvement in diabetes, obesity, and associated complications was confirmed by continuous oral supply of all three doses of CBP extracts (400, 200, and 100 mg/kg) for 84 days. Compared to the HFD control group, the significant inhibition of oxidative stress, activation of the antioxidant defense system, normalized activity of enzymes linked with sugar metabolism in the liver, and mRNA expression of genes associated with fat metabolism in the adipose tissue were confirmed in a dose-dependent manner in all CBP extract-administered groups. In particular, the CBP extract (200 mg/kg)-supplied group showed comparable effects to that of the metformin (250 mg/kg)-supplied group. Thus, it was judged that the administration of CBP extracts shows improvement effects for type 2 diabetes, obesity, and associated complications by regulating oxidative stress through the AMPK signaling pathway. Therefore, CBP extracts could be used to develop an effective functional food material or a therapeutic agent for type 2 diabetics and its associated complications, including diabetic nephropathy, NAFLD, and hyperlipidemia.

## Figures and Tables

**Table 1:** Changes in absolute organ weight in mice supplied with either NFD or HFD.

Groups	Absolute Organ Weights (g)				
	Liver	Kidney	Pancreas	Periovarian Fat Pads	Abdominal Wall Fat Pads
Controls					
NFD	1.238 ± 0.031	0.208 ± 0.011	0.264 ± 0.039	0.062 ± 0.035	0.035 ± 0.026
HFD	1.906 ± 0.094 ^a^	0.297 ± 0.020 ^a^	0.255 ± 0.021	0.452 ± 0.125 ^a^	0.387 ± 0.051 ^a^
Reference					
MET_250_	1.526 ± 0.102 ^ac^	0.241 ± 0.012 ^ac^	0.259 ± 0.026	0.237 ± 0.072 ^ac^	0.207 ± 0.024 ^ac^
Test materials					
CBP_400_	1.418 ± 0.144 ^bc^	0.227 ± 0.009 ^ac^	0.262 ± 0.026	0.163 ± 0.022 ^ac^	0.135 ± 0.039 ^ac^
CBP_200_	1.436 ± 0.081 ^ac^	0.241 ± 0.009 ^ac^	0.258 ± 0.012	0.236 ± 0.053 ^ac^	0.215 ± 0.068 ^ac^
CBP_100_	1.638 ± 0.097 ^ac^	0.258 ± 0.009 ^ac^	0.259 ± 0.023	0.301 ± 0.023 ^ad^	0.264 ± 0.043 ^ac^

Values are expressed as mean ± S.D. of 10 mice; NFD: Normal pellet diet; HFD: 45% Kcal high-fat diet; CBP: Coffee berry pulp extracts; DT3: Dunnett’s T3; NFD control: Vehicle (10 mL/kg distilled water) orally administered mice with NFD supply; HFD control: Vehicle (10 mL/kg distilled water) orally administered mice with HFD supply; MET: Metformin-administrated mice; ^a^ *p* < 0.01 and ^b^ *p* < 0.05 as compared with the NFD control using the DT3 test; ^c^ *p* < 0.01 and ^d^ *p* < 0.05 as compared with the HFD control using the DT3 test.

**Table 2:** Changes in relative organ weight in mice supplied with either NFD or HFD.

Groups	Relative Organ Weights (% of Body Weights)				
	Liver	Kidney	Pancreas	Periovarian Fat Pads	Abdominal Wall Fat Pads
Controls					
NFD	4.223 ± 0.219	0.709 ± 0.036	0.900 ± 0.129	0.211 ± 0.119	0.119 ± 0.091
HFD	4.393 ± 0.389	0.685 ± 0.071	0.589 ± 0.081 ^c^	1.030 ± 0.229 ^a^	0.889 ± 0.117 ^c^
Reference					
MET_250_	4.381 ± 0.238	0.692 ± 0.055	0.744 ± 0.076 ^e^	0.678 ± 0.109 ^ab^	0.595 ± 0.082 ^ce^
Test materials					
CBP_400_	4.259 ± 0.357	0.685 ± 0.059	0.789 ± 0.073 ^e^	0.491 ± 0.085 ^ab^	0.402 ± 0.105 ^ce^
CBP_200_	4.423 ± 0.235	0.693 ± 0.024	0.744 ± 0.047 ^de^	0.677 ± 0.134 ^ab^	0.618 ± 0.188 ^cf^
CBP_100_	4.400 ± 0.244	0.693 ± 0.025	0.694 ± 0.049 ^cf^	0.808 ± 0.074 ^ab^	0.710 ± 0.122 ^cf^

Values are expressed as mean ± S.D. of 10 mice; NFD: Normal pellet diet; HFD: 45% Kcal high-fat diet; CBP: Coffee berry pulp extracts; THSD: Tukey’s Honest Significant Difference; DT3: Dunnett’s T3; NFD control: Vehicle (10 mL/kg distilled water) orally administered mice with NFD supply; HFD control: Vehicle (10 mL/kg distilled water) orally administered mice with HFD supply; MET: Metformin-administrated mice; ^a^ *p* < 0.01 as compared with NFD control by THSD test; ^b^ *p* < 0.01 as compared with the HFD control using the THSD test; ^c^ *p* < 0.01 and ^d^ *p* < 0.05 as compared with the NFD control using the DT3 test; ^e^ *p* < 0.01 and ^f^ *p* < 0.05 as compared with the HFD control using the DT3 test.

**Table 3:** Changes in the histopathology–histomorphometry of the periovarian and abdominal wall deposited fat pads in mice supplied with either NFD or HFD.

Groups	Periovarian Fat Pads		Abdominal Wall Fat Pads	
	Thickness (mm)	Adipocyte Diameters (μm)	Thickness (mm)	Adipocyte Diameters (μm)
Controls				
NFD	1.52 ± 0.34	41.48 ± 11.19	1.70 ± 0.37	39.48 ± 10.14
HFD	4.76 ± 0.89 ^c^	120.55 ± 17.20 ^a^	5.97 ± 1.03 ^c^	105.97 ± 12.31 ^a^
Reference				
MET_250_	2.80 ± 0.37 ^ce^	64.00 ± 12.72 ^ab^	3.35 ± 0.57 ^ce^	60.26 ± 12.61 ^ab^
Test materials				
CBP_400_	2.29 ± 0.49 ^de^	47.58 ± 10.66 ^b^	2.71 ± 0.69 ^de^	52.35 ± 10.04 ^b^
CBP_200_	2.82 ± 0.38 ^ce^	64.01 ± 10.72 ^ab^	3.39 ± 0.57 ^ce^	61.86 ± 13.38 ^ab^
CBP_100_	3.18 ± 0.37 ^ce^	82.65 ± 10.10 ^ab^	4.13 ± 0.30 ^ce^	81.08 ± 11.37 ^ab^

Values are expressed as mean ± S.D. of 10 mice; NFD: Normal pellet diet; HFD: 45% Kcal high-fat diet; CBP: Coffee berry pulp extracts; THSD: Tukey’s Honest Significant Difference; DT3: Dunnett’s T3; NFD control: Vehicle (10 mL/kg distilled water) orally administered mice with NFD supply; HFD control: Vehicle (10 mL/kg distilled water) orally administered mice with HFD supply; MET: Metformin-administrated mice; ^a^ *p* < 0.01 as compared with the NFD control using the THSD test; ^b^ *p* < 0.01 as compared with the HFD control using the THSD test; ^c^ *p* < 0.01 and ^d^ *p* < 0.05 as compared with the NFD control using the DT3 test; ^e^ *p* < 0.01 as compared with the HFD control using the DT3 test.

**Table 4:** Changes in blood glucose levels and serum lipid content in mice supplied with either NFD or HFD.

Groups	Glucose (mg/dL)	Total Cholesterol (mg/dL)	Triglyceride (mg/dL)	Low Density Lipoprotein (mg/dL)	High Density Lipoprotein (mg/dL)
Controls					
NFD	85.60 ± 12.13	88.10 ± 16.72	74.50 ± 14.38	20.10 ± 3.96	94.90 ± 12.64
HFD	253.50 ± 36.18 ^c^	259.60 ± 38.58 ^c^	221.10 ± 40.06 ^c^	97.50 ± 14.40 ^a^	19.80 ± 10.71 ^a^
Reference					
MET_250_	161.00 ± 17.11 ^cd^	167.10 ± 26.04 ^cd^	148.30 ± 19.11 ^cd^	62.70 ± 12.15 ^ab^	52.20 ± 11.78 ^ab^
Test materials					
CBP_400_	115.10 ± 17.24 ^cd^	114.60 ± 22.17 ^d^	103.60 ± 27.19 ^d^	44.50 ± 11.13 ^ab^	68.60 ± 16.77 ^ab^
CBP_200_	161.80 ± 13.86 ^cd^	165.00 ± 21.41 ^cd^	148.60 ± 12.85 ^cd^	62.80 ± 11.79 ^ab^	51.80 ± 10.34 ^ab^
CBP_100_	192.90 ± 15.21 ^cd^	196.20 ± 13.55 ^cd^	171.40 ± 11.83 ^ce^	74.60 ± 11.46 ^ab^	43.90 ± 10.12 ^ab^

Values are expressed as mean ± S.D. of 10 mice; NFD: Normal pellet diet; HFD: 45% Kcal high-fat diet; CBP: Coffee berry pulp extracts; THSD: Tukey’s Honest Significant Difference; DT3: Dunnett’s T3; NFD control: Vehicle (10 mL/kg distilled water) orally administered mice with NFD supply; HFD control: Vehicle (10 mL/kg distilled water) orally administered mice with HFD supply; MET: Metformin-administrated mice; ^a^ *p* < 0.01 as compared with the NFD control using the THSD test; ^b^ *p* < 0.01 as compared with the HFD control using the THSD test; ^c^ *p* < 0.01 as compared with the NFD control using the DT3 test; ^d^ *p* < 0.01 and ^e^ *p* < 0.05 as compared with the HFD control using the DT3 test.

**Table 5:** Changes in serum AST, ALT, ALP, LDH, GGT, BUN, and Creatinine levels in mice supplied with either NFD or HFD.

Groups	AST (IU/L)	ALT (IU/L)	ALP (IU/L)	LDH (×10 IU/L)	GGT (IU/L)	BUN (mg/dL)	Creatinine (mg/dL)
Controls							
NFD	70.90 ± 13.77	40.90 ± 11.62	70.10 ± 16.93	69.95 ± 14.19	5.50 ± 1.27	32.60 ± 10.96	0.55 ± 0.24
HFD	189.50 ± 14.24 ^a^	146.30 ± 11.28 ^a^	199.10 ± 27.10 ^c^	359.41 ± 78.70 ^c^	20.10 ± 1.37 ^a^	132.10 ± 15.14 ^a^	2.06 ± 0.24 ^a^
Reference							
MET_250_	129.40 ± 14.26 ^ab^	93.20 ± 11.71 ^ab^	125.20 ± 14.53 ^cd^	186.30 ± 28.93 ^cd^	12.00 ± 1.63 ^ab^	82.00 ± 19.50 ^ab^	1.32 ± 0.23 ^ab^
Test materials							
CBP_400_	103.20 ± 10.46 ^ab^	76.90 ± 16.54 ^ab^	95.60 ± 20.19 ^d^	136.41 ± 30.98 ^cd^	8.90 ± 1.66 ^ab^	63.00 ± 17.83 ^ab^	0.93 ± 0.16 ^ab^
CBP_200_	129.50 ± 14.35 ^ab^	91.50 ± 18.11 ^ab^	124.10 ± 7.91 ^cd^	194.03 ± 34.42 ^cd^	11.90 ± 1.66 ^ab^	82.20 ± 19.97 ^ab^	1.31 ± 0.19 ^ab^
CBP_100_	147.20 ± 13.10 ^ab^	115.30 ± 10.17 ^ab^	145.80 ± 8.12 ^cd^	219.80 ± 25.99 ^cd^	15.40 ± 1.58 ^ab^	101.90 ± 14.61 ^ab^	1.57 ± 0.12 ^ab^

Values are expressed as mean ± S.D. of 10 mice; NFD: Normal pellet diet; HFD: 45% Kcal high-fat diet; CBP: Coffee berry pulp extracts; THSD: Tukey’s Honest Significant Difference; DT3: Dunnett’s T3; NFD control: Vehicle (10 mL/kg distilled water) orally administered mice with NFD supply; HFD control: Vehicle (10 mL/kg distilled water) orally administered mice with HFD supply; MET: Metformin-administrated mice; ^a^ *p* < 0.01 as compared with the NFD control using the THSD test; ^b^ *p* < 0.01 as compared with the HFD control using the THSD test; ^c^ *p* < 0.01 as compared with the NFD control using the DT3 test; ^d^ *p* < 0.01 as compared with the HFD control using the DT3 test.

**Table 6:** Changes in histopathology–histomorphometry of the liver and kidney in mice supplied with either NFD or HFD.

Groups	Liver Steatosis (%/mm^2^ of Hepatic Tissues)	Mean Hepatocyte Diameters (μm/cell)	Degenerative Renal Tubule Numbers (%)
Controls			
NFD	3.08 ± 1.91	14.26 ± 1.39	4.90 ± 2.56
HFD	78.64 ± 10.66 ^c^	28.12 ± 2.46 ^a^	78.60 ± 10.13 ^c^
Reference			
MET_250_	48.80 ± 10.31 ^cd^	21.18 ± 2.33 ^ab^	45.60 ± 11.89 ^cd^
Test materials			
CBP_400_	27.54 ± 15.48 ^cd^	18.48 ± 2.24 ^ab^	28.10 ± 11.86 ^cd^
CBP_200_	47.86 ± 10.14 ^cd^	21.20 ± 2.00 ^ab^	45.90 ± 12.72 ^cd^
CBP_100_	55.34 ± 11.63 ^cd^	22.86 ± 1.72 ^ab^	55.50 ± 11.66 ^cd^

Values are expressed as mean ± S.D. of 10 mice; NFD: Normal pellet diet; HFD: 45% Kcal high-fat diet; CBP: Coffee berry pulp extracts; THSD: Tukey’s Honest Significant Difference; DT3: Dunnett’s T3; NFD control: Vehicle (10 mL/kg distilled water) orally administered mice with NFD supply; HFD control: Vehicle (10 mL/kg distilled water) orally administered mice with HFD supply; MET: Metformin-administrated mice; ^a^ *p* < 0.01 as compared with the NFD control using the THSD test; ^b^ *p* < 0.01 as compared with the HFD control using the THSD test; ^c^ *p* < 0.01 as compared with the NFD control using the DT3 test; ^d^ *p* < 0.01 as compared with the HFD control using the DT3 test.

**Table 7:** Changes in liver lipid peroxidation and antioxidant defense systems in mice supplied with either NFD or HFD.

Groups	Lipid Peroxidation	Antioxidant Defense System		
	Malondialdehyde (nM/mg Tissue)	Glutathione (μM/mg Tissue)	Catalase (U/mg Tissue)	SOD (U/mg Tissue)
Controls				
NFD	10.52 ± 4.69	76.34 ± 10.49	70.53 ± 14.53	7.28 ± 1.14
HFD	87.95 ± 10.42 ^a^	11.21 ± 2.78 ^a^	11.42 ± 2.08 ^d^	1.04 ± 0.28 ^a^
Reference				
MET_250_	53.31 ± 12.12 ^ab^	34.16 ± 13.01 ^ab^	36.02 ± 11.70 ^df^	3.17 ± 0.90 ^ab^
Test materials				
CBP_400_	38.58 ± 11.52 ^ab^	46.61 ± 13.29 ^ab^	46.86 ± 10.86 ^ef^	4.21 ± 0.85 ^ab^
CBP_200_	53.62 ± 11.68 ^ab^	34.64 ± 13.86 ^ab^	35.86 ± 11.95 ^df^	3.16 ± 1.04 ^ab^
CBP_100_	64.47 ± 11.53 ^ab^	24.73 ± 11.88 ^a^	26.16 ± 10.20 ^dg^	2.33 ± 0.65 ^ac^

Values are expressed as mean ± S.D. of 10 mice; NFD: Normal pellet diet; HFD: 45% Kcal high-fat diet; CBP: Coffee berry pulp extracts; SOD: Superoxide dismutase; THSD: Tukey’s Honest Significant Difference; DT3: Dunnett’s T3; NFD control: Vehicle (10 mL/kg distilled water) orally administered mice with NFD supply; HFD control: Vehicle (10 mL/kg distilled water) orally administered mice with HFD supply; MET: Metformin-administrated mice; ^a^ *p* < 0.01 as compared with the NFD control using the THSD test; ^b^ *p* < 0.01 and ^c^ *p* < 0.05 as compared with the HFD control using the THSD test; ^d^ *p* < 0.01 and ^e^ *p* < 0.05 as compared with the NFD control using the DT3 test; ^f^ *p* < 0.01 and ^g^ *p* < 0.05 as compared with the HFD control using the DT3 test.

**Table 8:** Changes in hepatic glucose-regulating enzyme activities in mice supplied with either NFD or HFD.

Groups	Glucokinase (nM/min/mg Protein)	Glucose-6-Phosphatase (nM/min/mg Protein)	PEPCK (nM/min/mg Protein)
Controls			
NFD	5.99 ± 1.57	119.25 ± 26.51	1.87 ± 0.67
HFD	1.65 ± 0.20 ^a^	350.53 ± 104.60 ^a^	5.82 ± 1.17 ^a^
Reference			
MET_250_	3.16 ± 0.71 ^ac^	206.90 ± 28.45 ^ad^	3.39 ± 0.65 ^ac^
Test materials			
CBP_400_	3.99 ± 1.09 ^ac^	155.51 ± 20.98 ^bc^	2.48 ± 0.42 ^c^
CBP_200_	3.13 ± 0.57 ^ac^	204.28 ± 27.26 ^ad^	3.37 ± 0.49 ^ac^
CBP_100_	2.55 ± 0.67 ^ad^	239.16 ± 32.57 ^a^	4.01 ± 0.33 ^ad^

Values are expressed as mean ± S.D. of 10 mice; NFD: Normal pellet diet; HFD: 45% Kcal high-fat diet; CBP: Coffee berry pulp extracts; PEPCK: Phosphoenolpyruvate carboxykinase; DT3: Dunnett’s T3; NFD control: Vehicle (10 mL/kg distilled water) orally administered mice with NFD supply; HFD control: Vehicle (10 mL/kg distilled water) orally administered mice with HFD supply; MET: Metformin-administrated mice; ^a^ *p* < 0.01 and ^b^ *p* < 0.05 as compared with the NFD control using the DT3 test; ^c^ *p* < 0.01 and ^d^ *p* < 0.05 as compared with the HFD control using the DT3 test.

**Table 9:** Changes in lipid metabolism-related mRNA expressions in the livers of mice supplied with either NFD or HFD.

Groups	Hepatic Tissue (Relative to Control/GAPDH)		
	ACC1	AMPKα1	AMPKα2
Controls			
NFD	1.00 ± 0.05	1.00 ± 0.05	1.01 ± 0.04
HFD	5.46 ± 0.81 ^a^	0.24 ± 0.05 ^a^	0.23 ± 0.05 ^a^
Reference			
MET_250_	3.02 ± 0.68 ^ac^	0.50 ± 0.13 ^ac^	0.46 ± 0.09 ^ac^
Test materials			
CBP_400_	2.07 ± 0.85 ^bc^	0.75 ± 0.15 ^ac^	0.68 ± 0.15 ^ac^
CBP_200_	3.04 ± 0.81 ^ac^	0.50 ± 0.12 ^ac^	0.46 ± 0.13 ^ac^
CBP_100_	4.09 ± 0.35 ^ac^	0.43 ± 0.12 ^ad^	0.39 ± 0.11 ^ad^

Values are expressed as mean ± S.D. of 10 mice; NFD: Normal pellet diet; HFD: 45% Kcal high-fat diet; CBP: Coffee berry pulp extracts; GAPDH: Glyceraldehyde 3-phosphate dehydrogenase; DT3: Dunnett’s T3; NFD control: Vehicle (10 mL/kg distilled water) orally administered mice with NFD supply; HFD control: Vehicle (10 mL/kg distilled water) orally administered mice with HFD supply; MET: Metformin-administrated mice; ^a^ *p* < 0.01 and ^b^ *p* < 0.05 as compared with the NFD control using the DT3 test; ^c^ *p* < 0.01 and ^d^ *p* < 0.05 as compared with the HFD control using the DT3 test.

**Table 10:** Changes in lipid metabolism-related mRNA expressions in the adipose tissue of mice supplied with either NFD or HFD.

Groups	Control		Reference	Test materials—CBP		
	NFD	HFD	Metformin	400 mg/kg	200 mg/kg	100 mg/kg
Adipose tissue (Relative to control/GAPDH)						
Leptin	1.00 ± 0.08	8.24 ± 0.74 ^d^	4.38 ± 0.73 ^de^	2.96 ± 0.79 ^de^	4.40 ± 0.65 ^de^	6.21 ± 0.78 ^de^
UCP2	1.00 ± 0.07	0.24 ± 0.07 ^d^	0.52 ± 0.14 ^de^	0.70 ± 0.16 ^de^	0.52 ± 0.13 ^de^	0.43 ± 0.08 ^de^
Adiponectin	1.00 ± 0.08	0.18 ± 0.05 ^a^	0.40 ± 0.13 ^ab^	0.59 ± 0.12 ^ab^	0.40 ± 0.13 ^ab^	0.33 ± 0.07 ^ac^
C/EBPα	1.00 ± 0.04	3.95 ± 1.25 ^d^	2.03 ± 0.33 ^de^	1.49 ± 0.24 ^de^	2.04 ± 0.19 ^df^	2.39 ± 0.09 ^df^
C/EBPβ	1.00 ± 0.05	4.34 ± 0.85 ^d^	2.39 ± 0.54 ^de^	1.68 ± 0.27 ^de^	2.38 ± 0.54 ^de^	3.10 ± 0.29 ^df^
SREBP1c	1.00 ± 0.05	3.01 ± 0.45 ^d^	1.88 ± 0.25 ^de^	1.46 ± 0.24 ^de^	1.89 ± 0.30 ^de^	2.27 ± 0.19 ^de^
PPARα	1.00 ± 0.06	0.20 ± 0.04 ^d^	0.36 ± 0.07 ^de^	0.53 ± 0.14 ^de^	0.37 ± 0.10 ^de^	0.31 ± 0.05 ^de^
PPARγ	1.00 ± 0.05	7.26 ± 1.17 ^d^	4.33 ± 0.83 ^de^	2.66 ± 0.79 ^de^	4.34 ± 0.71 ^de^	5.04 ± 0.53 ^de^
FAS	1.00 ± 0.06	17.03 ± 2.26 ^d^	9.53 ± 2.12 ^de^	6.57 ± 2.51 ^de^	9.59 ± 1.46 ^de^	12.17 ± 1.23 ^de^

Values are expressed as mean ± S.D. of 10 mice; NFD: Normal pellet diet; HFD: 45% Kcal high-fat diet; CBP: Coffee berry pulp extracts; GAPDH: Glyceraldehyde 3-phosphate dehydrogenase; THSD: Tukey’s Honest Significant Difference; DT3: Dunnett’s T3; NFD control: Vehicle (10 mL/kg distilled water) orally administered mice with NFD supply; HFD control: Vehicle (10 mL/kg distilled water) orally administered mice with HFD supply; ^a^ *p* < 0.01 as compared with the NFD control using the THSD test; ^b^ *p* < 0.01 and ^c^ *p* < 0.05 as compared with the HFD control using the THSD test; ^d^ *p* < 0.01 as compared with the NFD control using the DT3 test; ^e^ *p* < 0.01 and ^f^ *p* < 0.05 as compared with the HFD control using the DT3 test.

## Data Availability

Data are contained within the article and Supplementary Materials.

## Supplementary Materials

The following supporting information can be downloaded at: https://www.mdpi.com/article/10.3390/antiox13010010/s1, Table S1: Composition of normal and high-fat diets used in this study; Table S2: Oligonucleotides for real time RT-PCR; Table S3: Changes in body weight and mean daily food consumption in mice supplied with either NFD or HFD; Table S4: Changes in histopathology–histomorphometry of pancreas in mice supplied with either NFD or HFD; Figure S1: HPLC analysis of standard chlorogenic acid (A) and chlorogenic acid detected in CBP extracts (B); Figure S2: Body weight changes in mice supplied with either NFD or HFD; Figure S3: Total body and abdominal fat densities in mice supplied with either NFD or HFD; Figure S4: Representative gross body mass and abdominal fat pads with whole body DEXA images taken from mice supplied with either NFD or HFD; Figure S5: Representative histological images of the adipocytes, taken from periovarian- and abdominal wall-deposited fat pads of mice supplied with either NFD or HFD; Figure S6: Representative histological images of the liver, taken from mice supplied with either NFD or HFD; Figure S7: Representative histological images of the kidney, taken from mice supplied with either NFD or HFD.
