# Supplementary material for: Validating the Health Benefits of Coffee Berry Pulp Extracts in Mice with High-Fat Diet-Induced Obesity and Diabetes
Source: Antioxidants (Basel). 2023 Dec 20;13(1):10. doi: 10.3390/antiox13010010 (PMC10812732; doi:10.3390/antiox13010010)
Supplement: Supplementary file 1 [file antioxidants-13-00010-s001.zip › antioxidants-2715448-supplementary.pdf]

antioxidants MDPI

Supplementary File

Validating the Health Benefits of Coffee Berry Pulp Extracts in Mice with High-Fat Diet-Induced Obesity and Diabetes

Khawaja Muhammad Imran Bashir [1,2,†], Joo Wan Kim [3,†], Hye-Rim Park [4,5], Jae-Kyoung Lee [6,7], Beom-Rak Choi [4], Jae-Suk Choi [1,*] and Sae-Kwang Ku [5,*]

[1] Department of Seafood Science and Technology, The Institute of Marine Industry, Gyeongsang National University, Tongyeong 53064, Republic of Korea; imran.bashir@lstme.org
[2] German Engineering Research and Development Center for Life Science Technologies in Medicine and Environment, Busan 46742, Republic of Korea
[3] Department of Companion Animal Health, Daegu Haany University, Gyeongsan 38610, Republic of Korea
[4] Nutracore Co., Ltd., Suwon 16514, Republic of Korea
[5] Department of Anatomy and Histology, College of Korean Medicine, Daegu Haany University, Gyeongsan 38610, Republic of Korea
[6] CNS Pharm Korea Co., Ltd., Seoul 04043, Republic of Korea
[7] Department of Food Regulatory Science, College of Science and Technology, Korea University Sejong Campus, Sejong 30019, Republic of Korea
* Correspondence: jsc1008@gnu.ac.kr (J.-S.C.); gucci200@dhu.ac.kr (S.-K.K.); Tel.: +82-55-772-9142 (J.-S.C.); +82-53-819-1549 (S.-K.K.)
† These authors contributed equally to this work.

Table S1. Composition of normal and high-fat diets used in this study.

**Compositions***	**Normal pellet diet**	**High-fat diet**
Ingredient (g/kg)		
Casein	200	200
L-Cystein	3	3
Corn starch	150	72.8
Sucrose	500	172.8
Cellulose	50	50
Soybean Oil	50	25
Lard	0	177.5
Mineral mixture	35	35
Vitamin mixture	10	10
Choline bitartrate	2	2
Energy (kcal/g)	4.00	4.73
Protein (% kcal)	20	20
Carbohydrate (% kcal)	64	35
Fat (% kcal)	16	45

*45%Kcal Fat pellet diet (Research Diet, New Brunswick, NJ, USA) was used as high-fat diet and normal rodents pellet diet (Purinafeed, Seungnam, Korea) was used as normal-fat pellet diet.

Table S2. Oligonucleotides for real time RT-PCR.

Target	5′ – 3′	Sequence	GenBank Accession Number
PPARα	Forward<br>Reverse	ATGCCAGTACTGCCGTTTTC<br>GGCCTTGACCTTGTTCATGT	NM_011144
PPARγ	Forward<br>Reverse	AGTGGAGACCGCCCAGG<br>GCAGCAGGTTGTCTTGGATGT	NM_001127330
Leptin	Sense<br>Antisense	CCAAAACCCTCATCAAGACC<br>GTCCAACTGTTGAAGAATGTCCC	NM_008493
UCP2	Sense<br>Antisense	CCGCATTGGCCTCTACGACTCT<br>CCCCGAAGGCAGAAGTGAAGTG	NM_011671
Adiponectin	Sense<br>Antisense	CCCAAGGGAACTTGTGCAGGTTGGATG<br>GTTGGTATCATGGTAGAGAAGAAAGCC	NM_009605
C/EBPα	Sense<br>Antisense	TGGACAAGAACAGCAACGAGTAC<br>CGGTCATTGTCACTGGTCAACT	NM_001287523
C/EBPβ	Sense<br>Antisense	AAGCTGAGCGACGAGTACAAGA<br>GTCAGCTCCAGCACCTTGTG	NM_001287739
SREBP1c	Sense<br>Antisense	AGCCTGGCCATCTGTGAGAA<br>CAGACTGGTACGGGCCACAA	XM_006532714
FAS	Sense<br>Antisense	GCTGCGGAAACTTCAGGAAAT<br>AGAGACGTGTCACTCCTGGACTT	NM_007988
ACC1	Sense<br>Antisense	GCCATTGGTATTGGGGCTTAC<br>CCCGACCAAGGACTTTGTTG	NM_133360
AMPKα1	Sense<br>Antisense	AAGCCGACCCAATGACATCA<br>CTTCCTTCGTACACGCAAAT	XM_011245321
AMPKα2	Sense<br>Antisense	GATGATGAGGTGGTGGA<br>GCCGAGGACAAAGTGC	NM_178143
GAPDH	Sense<br>Antisense	CATCTTCCAGGAGCGAGACC<br>TCCACCACCCTGTTGCTGTA	NM_008084

RT-PCR: Reverse transcription polymerase chain reaction; PPAR: Peroxisome proliferator-activated receptor; UCP: Mitochondrial uncoupling protein; C/EBP: CCAAT-enhancer-binding protein; SREBP: Sterol regulatory element-binding protein; ACC1: Acetyl-CoA carboxylase 1; FAS: Fatty acid synthase; AMPK: 5' adenosine monophosphate-activated protein kinase; GAPDH: Glyceraldehydes 3-phosphate dehydrogenase.

Table S3. Changes in body weight and mean daily food consumption in mice supplied with either NFD or HFD.

**Groups**	**Body weights (g) at days after initial test substance treatment**				**Body weight gains during**		**Mean Daily Food Consumption** (g)
	8 days before [A]	1 day before [B]	0 day* [C]	84 days* [D]	Adapt period [B-A]	Administration period [D-C]	
Controls							
NFD	27.59 ± 0.89	28.19 ± 1.10	25.16 ± 1.18	29.37 ± 1.50	0.60 ± 0.49	4.21 ± 0.94	5.22 ± 0.58
HFD	27.81 ± 1.04	31.48 ± 1.86 [a]	28.70 ± 1.94 [a]	43.62 ± 3.30 [b]	3.67 ± 1.35 [a]	14.92 ± 3.10 [b]	4.38 ± 0.35 [b]
Reference							
MET_{250}	27.87 ± 1.16	31.44 ± 1.81 [a]	28.57 ± 1.69 [a]	34.86 ± 2.01 [bc]	3.57 ± 1.29 [a]	6.29 ± 1.15 [bc]	4.44 ± 0.33 [b]
Test materials							
CBP_{400}	27.71 ± 1.18	31.27 ± 1.34 [a]	28.57 ± 1.34 [a]	33.29 ± 2.08 [bc]	3.56 ± 1.19 [a]	4.72 ± 1.40 [c]	4.43 ± 0.37 [b]
CBP_{200}	27.59 ± 1.25	31.18 ± 1.29 [a]	28.18 ± 1.30 [a]	34.75 ± 1.41 [bc]	3.59 ± 1.08 [a]	6.57 ± 1.34 [bc]	4.47 ± 0.46 [b]
CBP_{100}	27.62 ± 0.96	31.26 ± 1.24 [a]	28.34 ± 1.03 [a]	37.24 ± 1.21 [bc]	3.64 ± 0.94 [a]	8.90 ± 1.12 [cd]	4.44 ± 0.29 [b]

Values are expressed as means ± S.D. of 10 mice; NFD: Normal pellet diet; HFD: 45%Kcal high-fat diet; MET: Metformin; CBP: Coffee berry pulp extracts; THSD: Tukey's Honest Significant Difference; DT3: Dunnett's T3; NFD control: Vehicle (10 mL/kg distilled water) orally administered mice with NFD supply; HFD control: Vehicle (10 mL/kg distilled water) orally administered mice with HFD supply; *All animals were overnight fasted; [a] $p < 0.01$ as compared with NFD control by THSD test; [b] $p < 0.01$ as compared with NFD control by DT3 test; [c] $p < 0.01$ and [d] $p < 0.01$ as compared with HFD control by DT3 test.

Table S4. Changes in histopathology-histomorphometry of pancreas in mice supplied with either NFD or HFD.

Groups	**Zymogen granules** (%/mm² of exocrine)	**Mean islet numbers** (numbers/10 mm²)	**Mean islet diameter** (μm/islet)	**Insulin-IR cells** (cells/ mm²) [A]	**Glucagon-IR cells** (cells/ mm²) [B]	**Insulin/glucagon ratio** [A/B]
Controls						
NFD	58.16 ± 10.03	5.80 ± 1.48	106.95 ± 12.90	136.40 ± 13.36	39.40 ± 8.85	3.54 ± 0.45
HFD	14.88 ± 5.88 [a]	18.70 ± 2.71 [a]	227.60 ± 19.65 [a]	2857.50 ± 217.86 [c]	445.70 ± 29.40 [c]	6.41 ± 0.22 [c]
Reference						
MET_{250}	37.24 ± 10.79 [ab]	10.00 ± 1.89 [ab]	157.67 ± 18.08 [ab]	1433.50 ± 375.75 [cd]	287.40 ± 57.01 [cd]	4.97 ± 0.63 [cd]
Test materials						
CBP_{400}	47.19 ± 10.55 [b]	7.90 ± 1.29 [b]	134.74 ± 13.45 [ab]	649.30 ± 266.85 [cd]	153.80 ± 49.48 [cd]	3.91 ± 0.75 [d]
CBP_{200}	37.56 ± 11.75 [ab]	10.50 ± 1.51 [ab]	156.46 ± 15.21 [ab]	1392.20 ± 362.98 [cd]	286.20 ± 70.98 [cd]	4.86 ± 0.33 [cd]
CBP_{100}	31.83 ± 10.90 [ab]	12.60 ± 1.78 [ab]	180.04 ± 14.34 [ab]	1958.00 ± 341.04 [cd]	364.90 ± 51.50 [cd]	5.37 ± 0.54 [cd]

Values are expressed as means ± S.D. of 10 mice; NFD: Normal pellet diet; HFD: 45%Kcal high-fat diet; MET: Metformin; CBP: Coffee berry pulp extracts; IR: Immunoreactive; THSD: Tukey's Honest Significant Difference; DT3: Dunnett's T3; NFD control: Vehicle (10 mL/kg distilled water) orally administered mice with NFD supply; HFD control: Vehicle (10 mL/kg distilled water) orally administered mice with HFD supply; [a] $p < 0.01$ as compared with NFD control by THSD test; [b] $p < 0.01$ as compared with HFD control by THSD test; [c] $p < 0.01$ as compared with NFD control by DT3 test; [d] $p < 0.01$ as compared with HFD control by DT3 test.

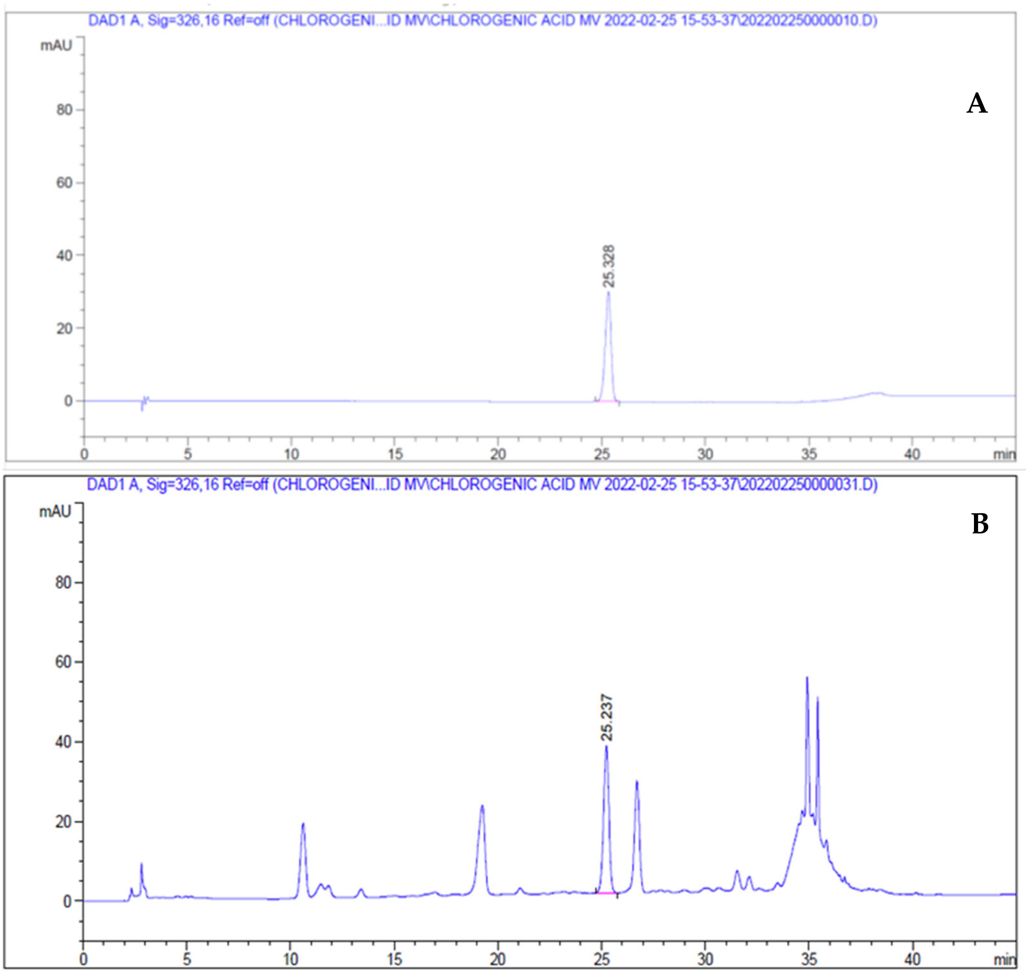

Figure S1. HPLC analysis of standard chlorogenic acid (**A**) and chlorogenic acid detected in CBP extracts (**B**).

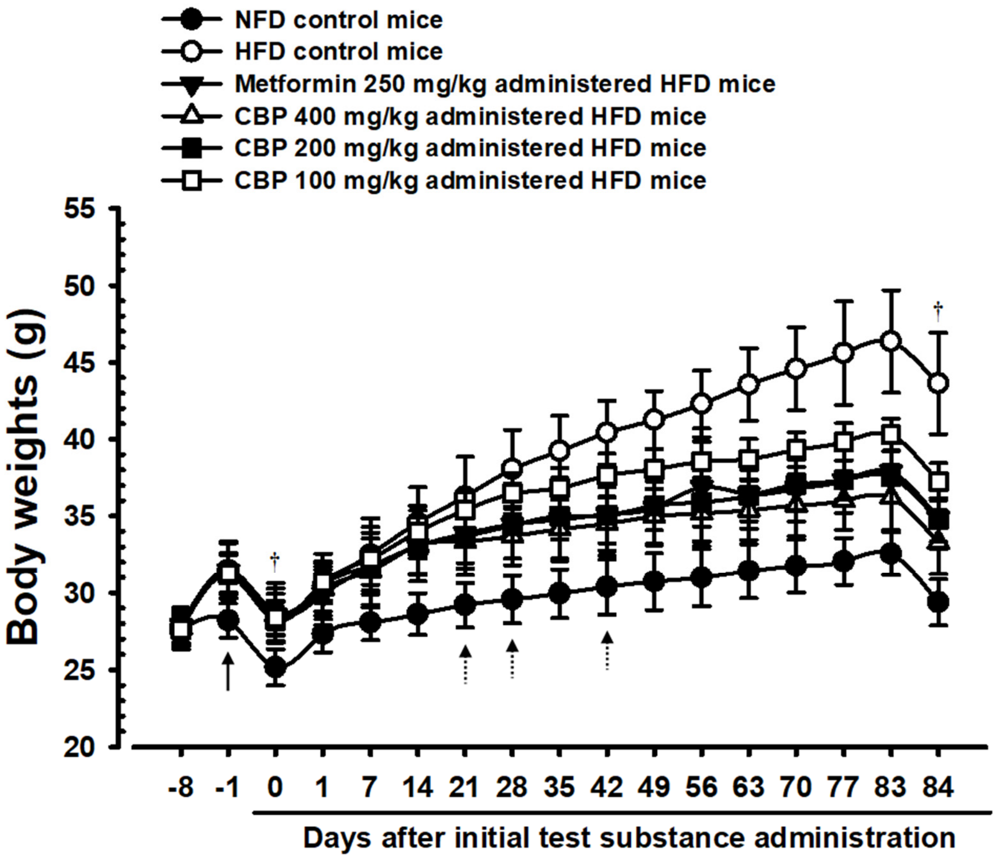

Figure S2. Body weight changes in mice supplied with either NFD or HFD.

Significant ($p < 0.01$) increases in body weight were observed in the HFD control mice compared to the NFD control mice 6 days after the HFD supply (indicated by **Arrows**). However, significant ($p < 0.01$; $p < 0.05$) decreases in body weight were detected mice treated with CBP extracts (400, 200, and 100 mg/kg) 21, 28, and 42 days after the initial administration and 28 days after the initial administration of metformin (250 mg/kg), respectively, in comparison to the HFD control (indicated by **Dotted arrows**). Values are expressed as means ± S.D. of 10 mice; NFD: Normal pellet diet; HFD: 45%Kcal high-fat diet; CBP: Coffee berry pulp extracts; NFD control: Vehicle (10 mL/kg distilled water) orally administered mice with NFD supply; HFD control: Vehicle (10 mL/kg distilled water) orally administered mice with HFD supply; All animals were fasted overnight before the initial test substance administration and sacrifice (**†**); Day-8: Eight days before the initial test material administration; Day 0: The day of the initial test material administration; Day 84: Twenty-four hours after the last (84th) test material administration.

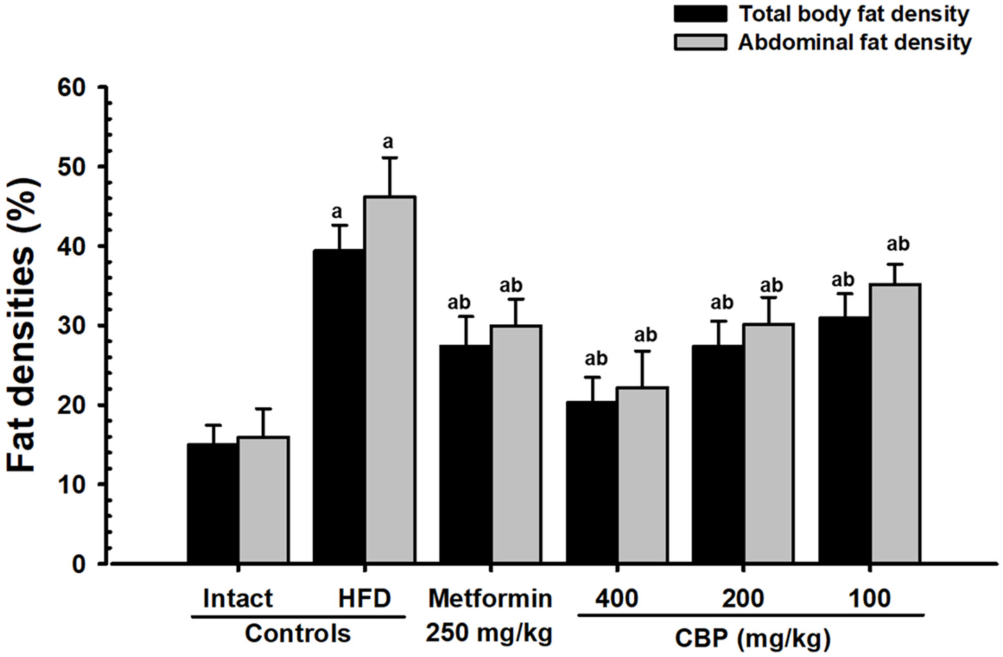

Figure S3. Total body and abdominal fat densities in mice supplied with either NFD or HFD.

Values are expressed as means ± S.D. of 10 mice; NFD: Normal pellet diet; HFD: 45%Kcal high-fat diet; CBP: Coffee berry pulp extracts; THSD: Tukey's Honest Significant Difference; NFD control: Vehicle (10 mL/kg distilled water) orally administered mice with NFD supply; HFD control: Vehicle (10 mL/kg distilled water) orally administered mice with HFD supply; [a] $p < 0.01$ as compared with NFD control by THSD test; [b] $p < 0.01$ as compared with HFD control by THSD test.

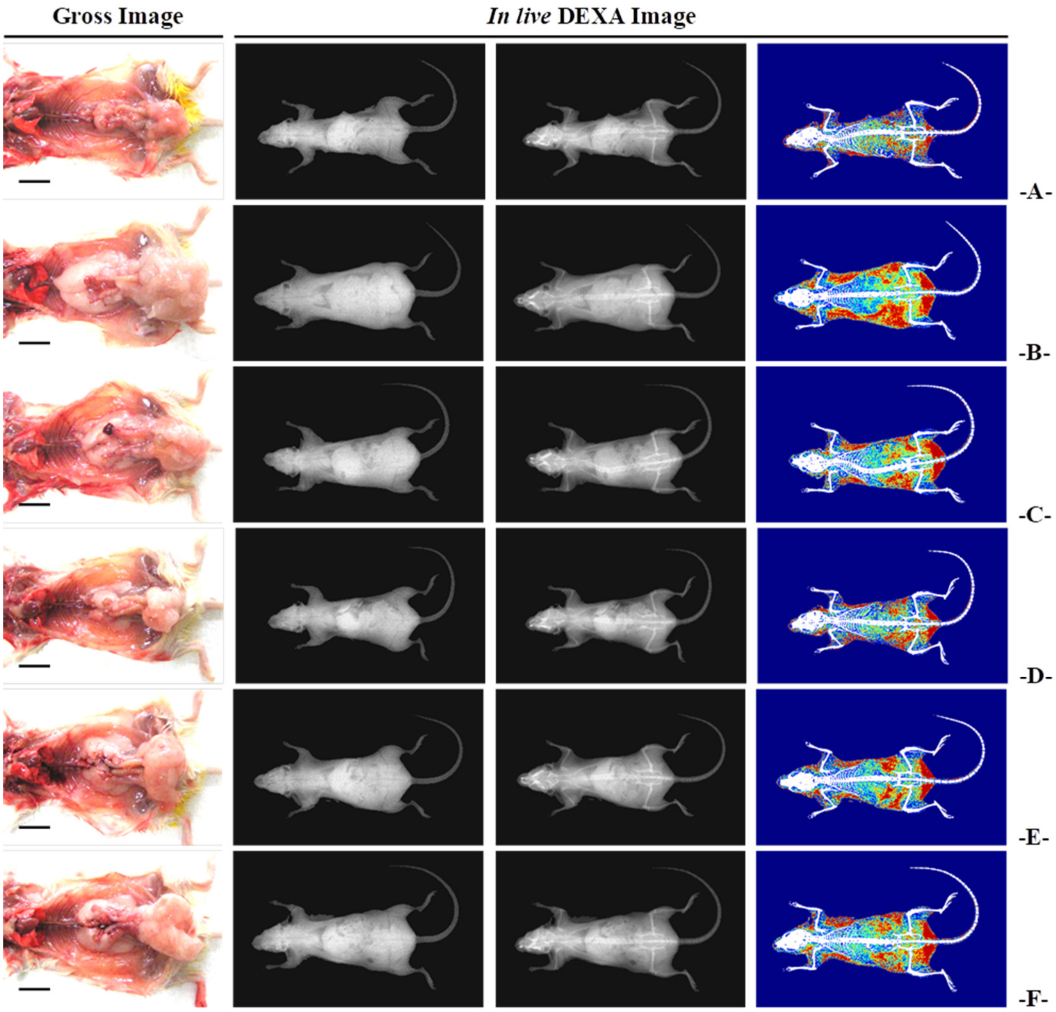

Figure S4. Representative gross body mass and abdominal fat pads with whole body DEXA images taken from mice supplied with either NFD or HFD.

A: Vehicle (10 mL/kg distilled water) orally administered mice with NFD supply (NFD control); **B**: Vehicle (10 mL/kg distilled water) orally administered mice with HFD supply (HFD control); **C**: Metformin (250 mg/kg) orally administered mice with HFD supply (MET_{250}); **D**: CBP (400 mg/kg) orally administered mice with HFD supply (CBP_{400}); **E**: CBP (200 mg/kg) orally administered mice with HFD supply (CBP_{200}); **F**: CBP (100 mg/kg) orally administered mice with HFD supply (CBP_{100}); NFD: Normal pellet diet; HFD: 45%Kcal high-fat diet; CBP: Coffee berry pulp extracts; DEXA: Dual-energy x-ray absorptiometry; Scale bar: 12.00 mm.

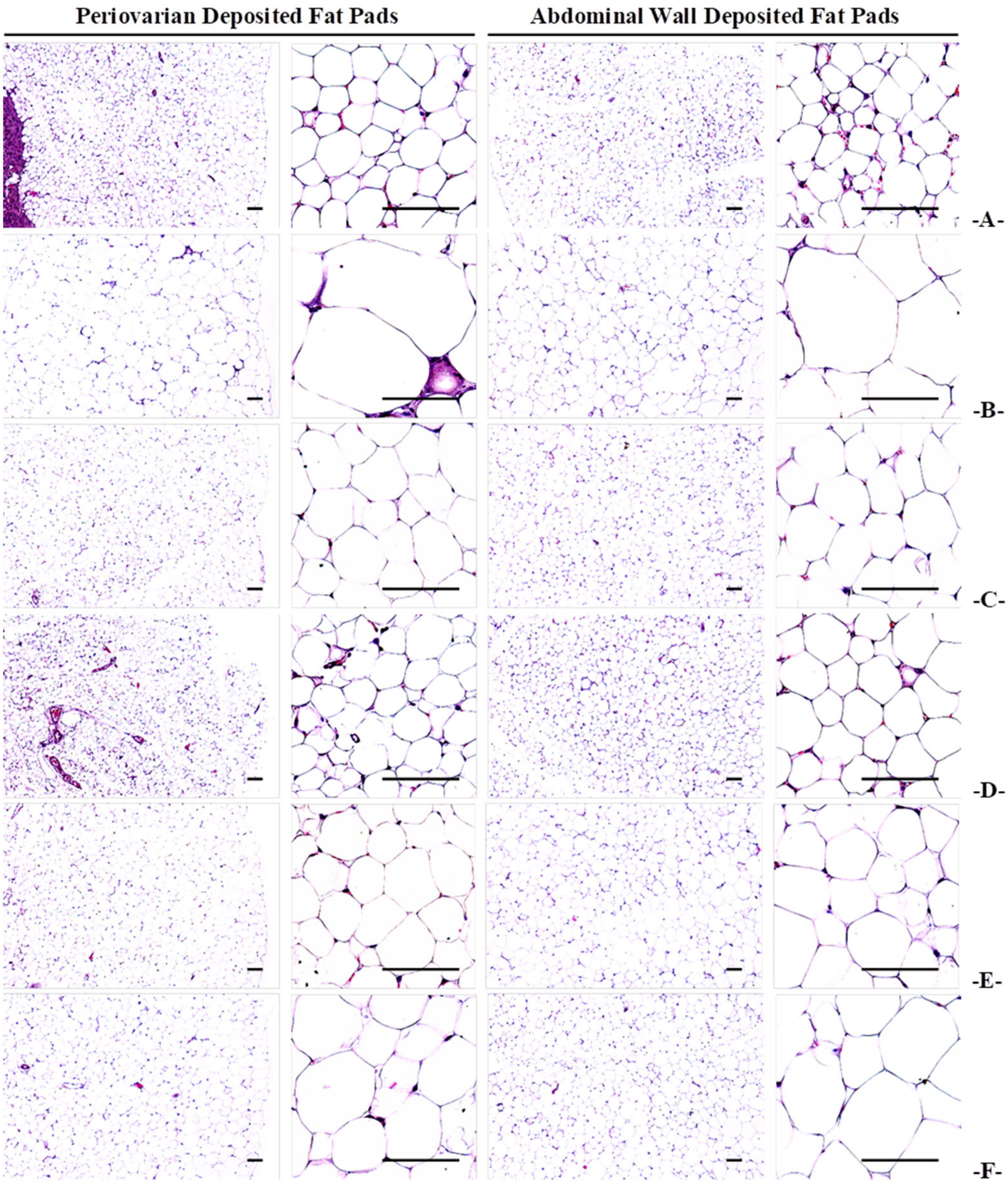

Figure S5. Representative histological images of the adipocytes, taken from periovarian and abdominal wall deposited fat pads of mice supplied with either NFD or HFD.

A: Vehicle (10 mL/kg distilled water) orally administered mice with NFD supply (NFD control); **B**: Vehicle (10 mL/kg distilled water) orally administered mice with HFD supply (HFD control); **C**: Metformin (250 mg/kg) orally administered mice with HFD supply (MET_{250}); **D**: CBP extracts (400 mg/kg) orally administered mice with HFD supply (CBP_{400}); **E**: CBP extracts (200 mg/kg) orally administered mice with HFD supply (CBP_{200}); **F**: CBP extracts (100 mg/kg) orally administered mice with HFD supply (CBP_{100}); NFD: Normal pellet diet; HFD: 45%Kcal high-fat diet; CBP: Coffee berry pulp extract; All samples were stained with hematoxylin and eosin; Scale bars: 80 μm.

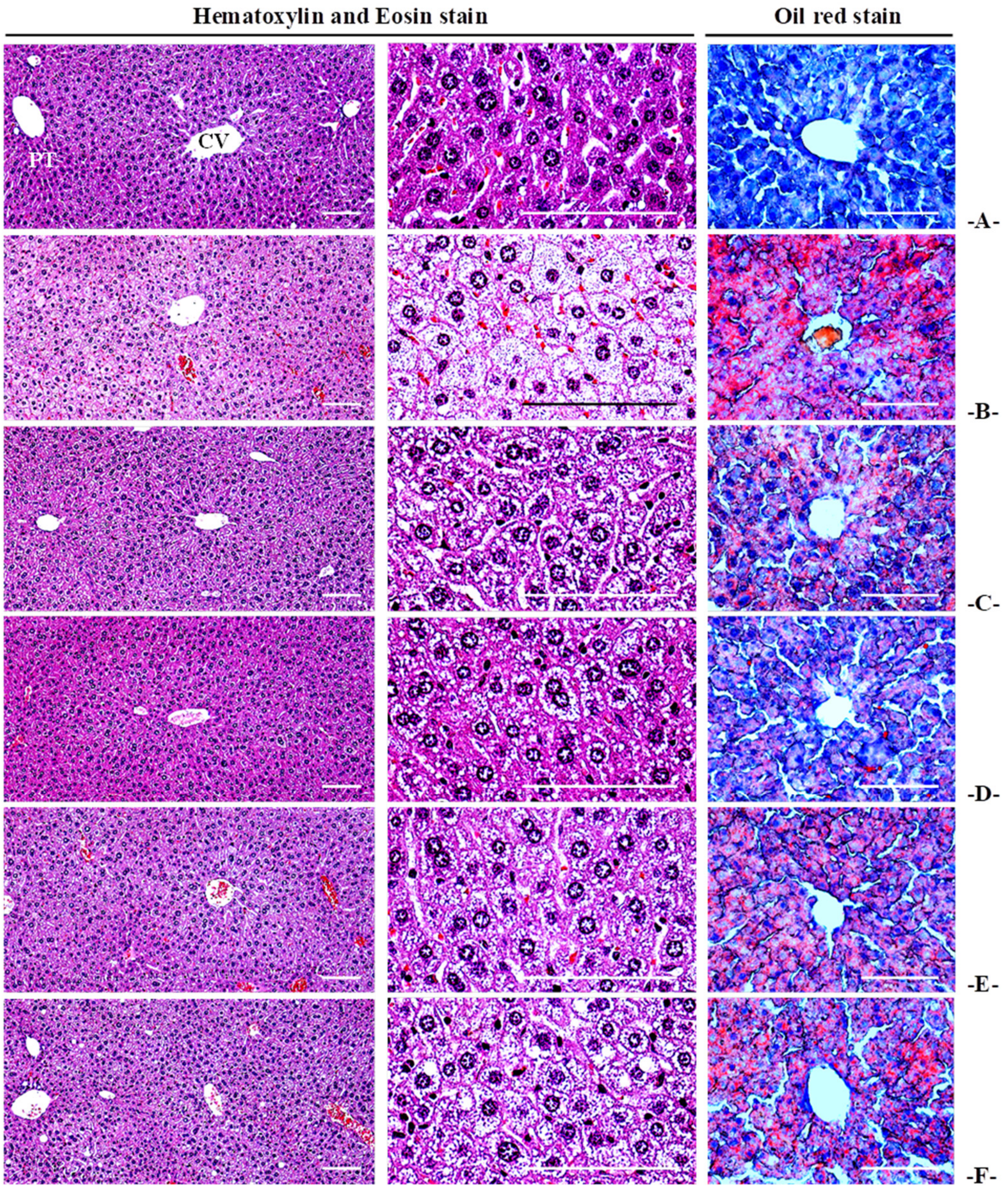

Figure S6. Representative histological images of the liver, taken from mice supplied with either NFD or HFD.

A: Vehicle (10 mL/kg distilled water) orally administered mice with NFD supply (NFD control); **B**: Vehicle (10 mL/kg distilled water) orally administered mice with HFD supply (HFD control); **C**: Metformin (250 mg/kg) of metformin oral administered mice with HFD supply (MET_{250}); **D**: CBP extracts (400 mg/kg) orally administered mice with HFD supply (CBP_{400}); **E**: CBP extracts (200 mg/kg) orally administered mice with HFD supply (CBP_{200}); **F**: CBP extracts (100 mg/kg) orally administered mice with HFD supply (CBP_{100}); CV: Central vein; NFD: Normal pellet diet; HFD: 45%Kcal high-fat diet; CBP: Coffee berry pulp extract; Scale bars: 80 μm.

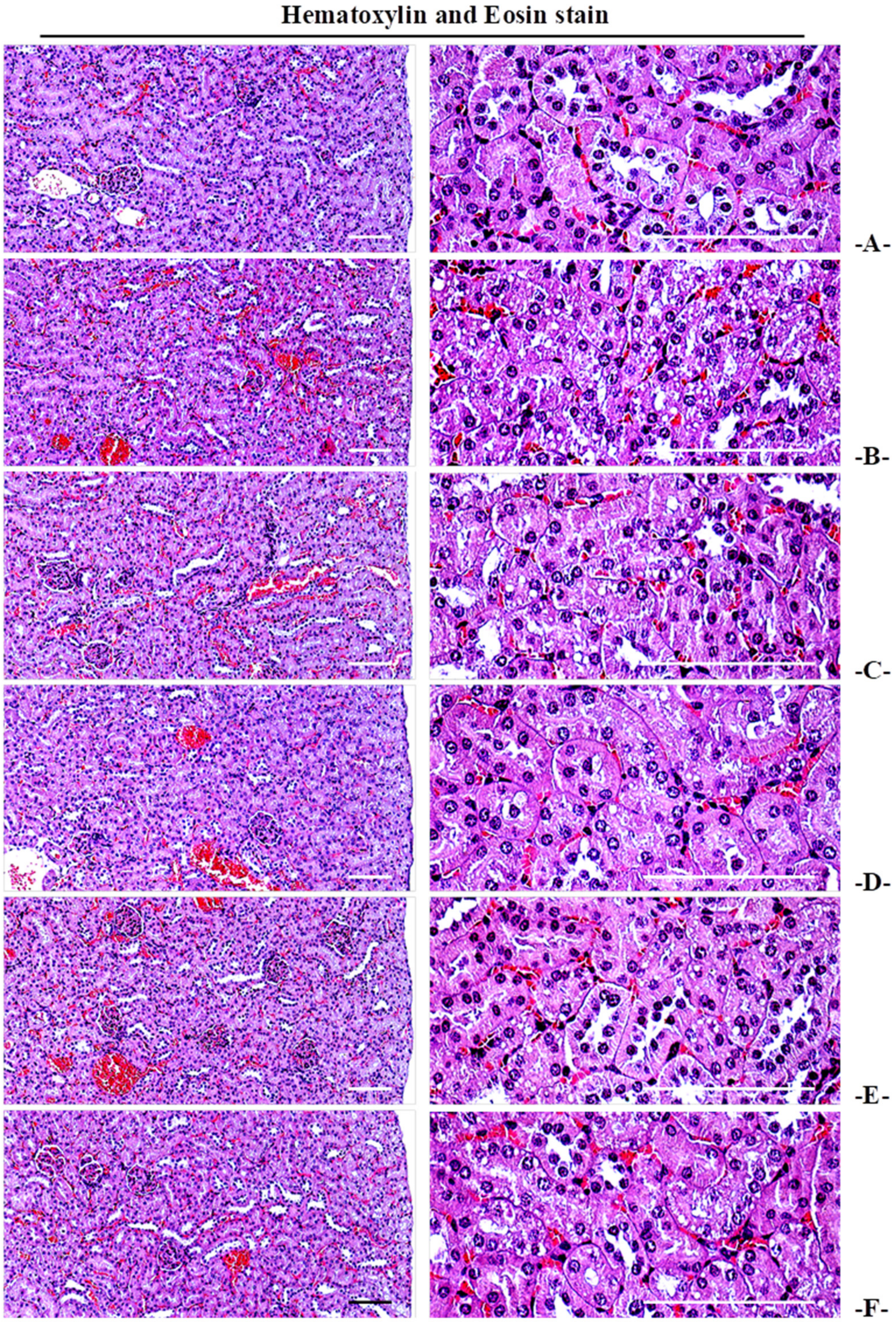

Figure S7. Representative histological images of the kidney, taken from mice supplied with either NFD or HFD.

A: Vehicle (10 mL/kg distilled water) orally administered mice with NFD supply (NFD control); **B**: Vehicle (10 mL/kg distilled water) orally administered mice with HFD supply (HFD control); **C**: Metformin (250 mg/kg) of metformin oral administered mice with HFD supply (MET_{250}); **D**: CBP extracts (400 mg/kg) orally administered mice with HFD supply (CBP_{400}); **E**: CBP extracts (200 mg/kg) orally administered mice with HFD supply (CBP_{200}); **F**: CBP extracts (100 mg/kg) orally administered mice with HFD supply (CBP_{100}); NFD: Normal pellet diet; HFD: 45%Kcal high-fat diet; CBP: Coffee berry pulp extract; All samples were stained with hematoxylin and eosin; Scale bars: 80 μm.
